# Accelerated seed dispersal along linear disturbances in the Canadian oil sands region

**DOI:** 10.1038/s41598-018-22678-y

**Published:** 2018-03-19

**Authors:** David Roberts, Simone Ciuti, Quinn E. Barber, Caitlin Willier, Scott E. Nielsen

**Affiliations:** 1 Department of Geography, University of Calgary, ES-356, 2500 University Drive NW, Calgary, Alberta T2N 1N4 Canada; 2 Arctic Institute of North America, University of Calgary, ES-1040, 2500 University Drive NW, Calgary, Alberta T2N 1N4 Canada; 3 School of Biology and Environmental Science, University College Dublin, Science West, Belfield, D4, Dublin, Ireland; 4 Department of Renewable Resources, University of Alberta, 705 General Services Building, Edmonton, Alberta T6G 2H1 Canada

## Abstract

Habitat fragmentation is typically seen as inhibiting movement via erosion in connectivity, although some patterns of early-phase disturbance, such as narrow linear disturbances in otherwise undisturbed forests, may actually facilitate the dispersal of certain species. Such features are common in Alberta’s oil sands region as legacies from seismic hydrocarbon exploration used to map oil reserves. Many of the ecological implications of these disturbances are unknown. Here, we investigate the effect of these forest dissections by experimentally testing dispersal patterns along seismic lines compared with adjacent forests using two proxy materials for wind-dispersed seeds, *Typha latifolia* seed and goose down feathers. We found that wind speeds were up to seven times higher and 95^th^ percentile seed dispersal distances nearly four times farther on seismic lines compared with undisturbed forests and the corresponding effect of these features on seed dispersal distances can be substantial, potentially facilitating future changes in composition and ecological processes in boreal forests. This raises important considerations for native and invasive species, particularly in the context of climate change and the associated importance of seed movement and migration.

## Introduction

Forest fragmentation is considered one of the main threats to biodiversity^1^ altering pre-disturbance processes^2^, decreasing genetic diversity of species due to isolation of populations^3^, changing species behaviours and dynamics, and promoting the introduction of non-native species^4^. While we generally think of fragmentation as landscapes composed of small remnant habitats within a matrix of highly altered anthropogenic land uses potentially connected via narrow bands of intact habitat, such as agricultural or urban areas^5,6^, the opposite pattern also exists. Early in the process of fragmentation, intact habitats are often disturbed with only a few initial patches or linear incisions^7^. When these first disturbances are narrow but long clearings, initial biological responses are likely to be alterations of the movement and behaviour of organisms. Thus, despite their small footprint, these dissections within otherwise relatively intact habitats can have profound effects on the dynamics and composition of species assemblages.

Much of our understanding of the ecological effects of linear dissections comes from research on anthropogenic features such as roads^8,9^. Among other effects, road clearings increase the intensity and turbidity of local wind patterns and, as such, facilitate airborne seed dispersal^10^. One could anticipate that even narrow linear disturbances can increase wind speeds, wind turbulence, and availability of colonization sites (i.e. without an impenetrable paved surface), thus accelerating the spread of wind-dispersed plants. However, seed dispersal is complex and dependent on many factors including the aerodynamic properties of diaspores, the height from which they released, and the quality of the microsites onto which they are deposited^11^. Among primarily wind dispersed seed, wind velocities are generally understood to be a principal factor determining dispersal distances^12,13^, presenting the potential for linear dissections to redirect and accelerate winds, thus increasing effective seed dispersal distances^14^.

**Figure 1:**
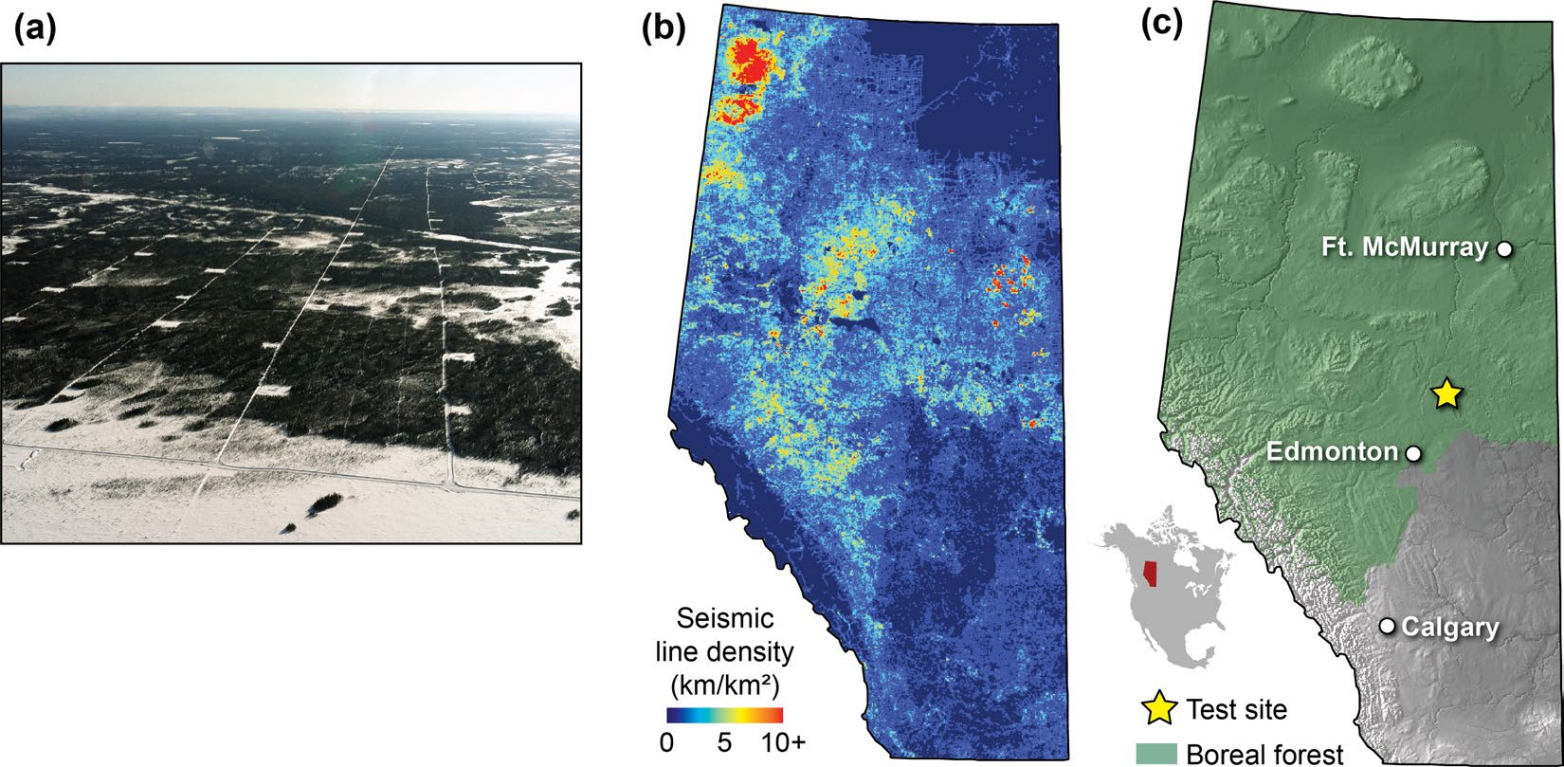
Seismic lines near our study site. (**a**) Aerial photo of seismic lines in the Alberta boreal forest (photo: Fiera Biological Consulting, used with permission). (**b**) Map of seismic line density (km/km²) in the province of Alberta^17^. (**c**) Study area map showing our study site within the province of Alberta. The extent of boreal forest coverage is shown in green^50^. Maps were created in ESRI ArcMap v10.4 (http://www.esri.com/), the R program for statistical computing^41^, and Adobe Illustrator CC 2015 (http://www.adobe.com/products/illustrator.html).

In many forested regions, a major source of such dissections is the manual clearing of forests for seismic exploration, common in the early phases of hydrocarbon extraction. These linear clearings, known as “seismic lines”, vary in width between two and eight metres and, in addition to more traditional linear developments such as roads, railways, power lines, and pipelines, are now the largest contributor to forest fragmentation in the Canadian boreal forest in northern Alberta (Fig. 1a). In this region, seismic line densities average 1.5 km/km^2^ and often reach 10 km/km^2^ in some locations with more extensive exploration^15,16^, or even up to 40 km/km^2^ in the most disturbed areas^17^ (Fig. 1b). By comparison, the average density of roads in the same region is 0.1 km/km² with a maximum density of 3.2 km/km² ^17^. In many cases, these seismic line footprints show little sign of recovery to a pre-disturbance state, with approximately one-third failing to recover even 50 years after the initial disturbance^15,18^. These changes alter the behaviour of birds^19^ and mammals^20^, including notable species of concern such as woodland caribou^21^, with particular implications for predator-prey dynamics^22,23^. Plant communities have also been found to change along linear disturbances, with differences persisting even after decades of natural recovery^24^. Although these forest dissections are known to act similarly to ecological corridors by increasing movements of mammals^20,23^, their potential to alter seed dispersal patterns by acting as vectors of dispersal in plants (i.e. anthropogenic corridors) has received little attention. Further, it may be difficult to generalise seed dispersal effects to seismic lines from other linear forest clearings, whether naturally occurring (e.g. rivers) or anthropogenic (e.g. roads), due to transformed ground cover or other transport vectors such as water or humans.

From a conservation perspective, increases in seed dispersal can have both positive and negative effects on communities. Given that species need to track suitable climatic habitats into the future^25,26^, facilitating dispersal can increase species’ potential for climate change adaptation via increased migration rates, which may be an even more effective process for maintaining genetic connectivity than gene flow through pollen transfer^27^. By the same mechanisms, however, increased potential for seed dispersal would also facilitate the undesirable spread of wind-dispersed invasive species that may threaten other species. This has been demonstrated, for example, by a significant increase in non-native plant species in association with silvicultural disturbance in the boreal forest^28^.

To properly investigate the potential ecological risks and benefits of these changes, a better understanding of the effects of incisions and dissections on seed dispersal is required. In this study, we aim to assess dispersal patterns of primarily wind-dispersed seed along seismic lines in an otherwise forested landscape. We expect dispersal along seismic lines to be higher than in undisturbed forests due to increased wind speeds, we focus here on quantifying the magnitude (effect size) of this difference, which is largely unknown. We test for these differences at a natural upland forest test site in northern Alberta, Canada (Fig. 1c), by measuring experimental dispersal rates of two proxies for wind-dispersed seed, commercial goose down feathers and seeds of *Typha latifolia* (common cattail; hereafter *Typha*), to minimize our effects of the experiment on the site. To confirm that the wind dispersal of our proxies is representative of other species, we perform laboratory loft tests on several local species. Further, to assess the generalisability of the specific conditions at our sites during our experimental trials, we compare experimental wind conditions with general wind trends throughout the region. Our results are discussed in the larger context of landscape-scale conservation, invasive species management, and climate change adaptation.

## Results

### Drivers of seed dispersal

**Figure 2:**
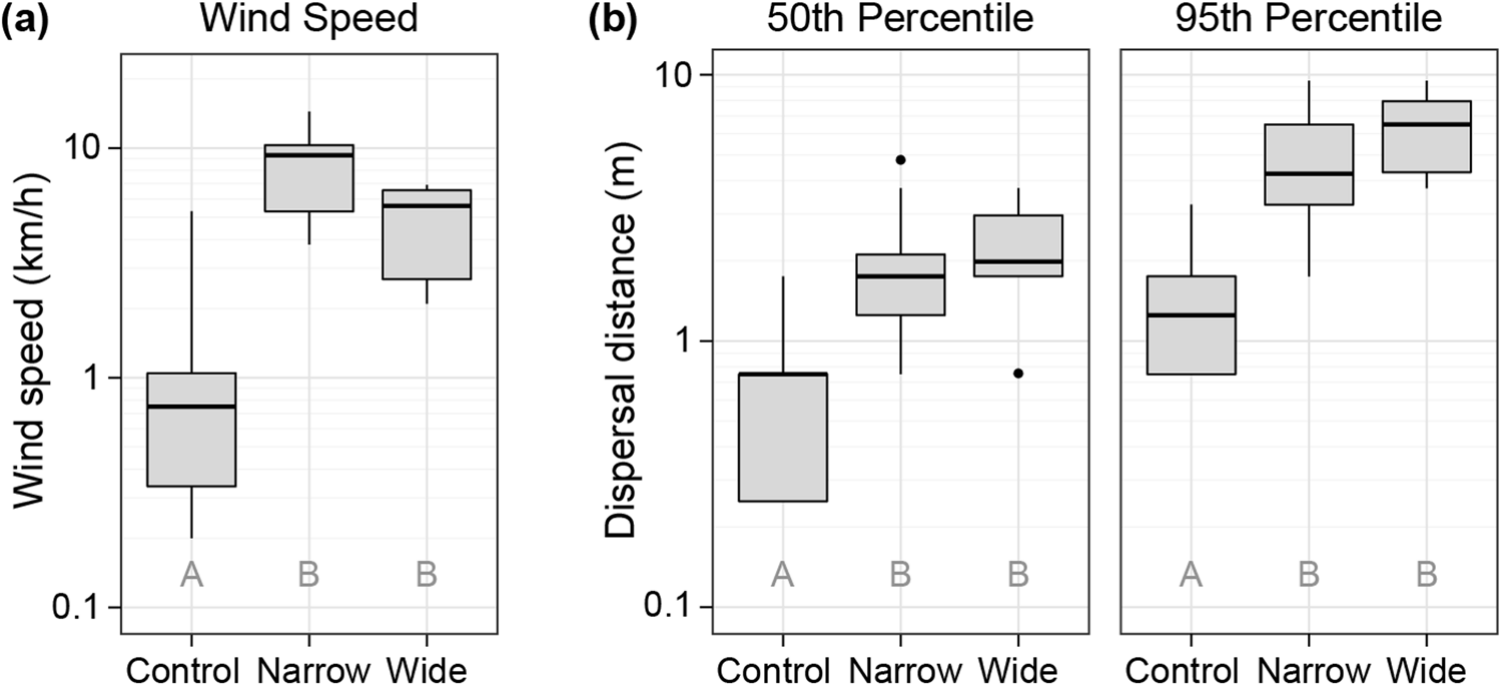
Wind speeds and dispersal distances on different sites. Observed differences in (**a**) wind speeds and (**b**) dispersal distances between the control forests (Control), seismic lines 5.3 to 6 m wide (Narrow), and seismic lines 7.5 to 8 m wide (Wide). Within each plot, different letters indicate that groups are significantly different at p < 0.001 (see text for details). Boxes represent interquartile ranges, whiskers extend to data ranges minus outliers (>1.5 interquartile range, shown as dots), and thick bars represent medians. Note that y-axes are log10 scaled to facilitate visualisation.

Overall, wind speeds during experimental tests (Fig. 2a) were seven times higher on seismic lines (n* = *12, mean = 7.7 km/hr, SD = 3.8 km/h) compared with adjacent control forests (n* = *12, mean = 1.1 km/hr, SD = 1.4 km/h), a difference determined to be statistically significant with a two-sample t-test (t = −7.04, df = 17.4, p = 1.73e-6). When we compared wind speeds by line width category (control forest vs. narrow seismic vs. wide seismic) in an analysis of variance (ANOVA), we found a significant main effect of line width (F_2,22_ = 25.61, p = 2.33e-6). Multiple comparisons with Tukey’s HSD test indicated that, while the difference between narrow lines (n = 3) and control forests (n = 12) (Δ means = 7.5 km/hr, adjusted p = 2.10e-6) or that of wide lines (n = 9) and control forests (Δ means = 3.8 km/hr, adjusted p = 4.35e-3) were both significant, the difference between narrow lines and wide lines was not (Δ means = 3.7, adjusted p = 0.526). Only a single wind speed measurement in the control forests (5.3 km/h) was within the ranges of all wind speeds measured on the seismic lines (2.1 to 14.4 km/h).

**Figure 3:**
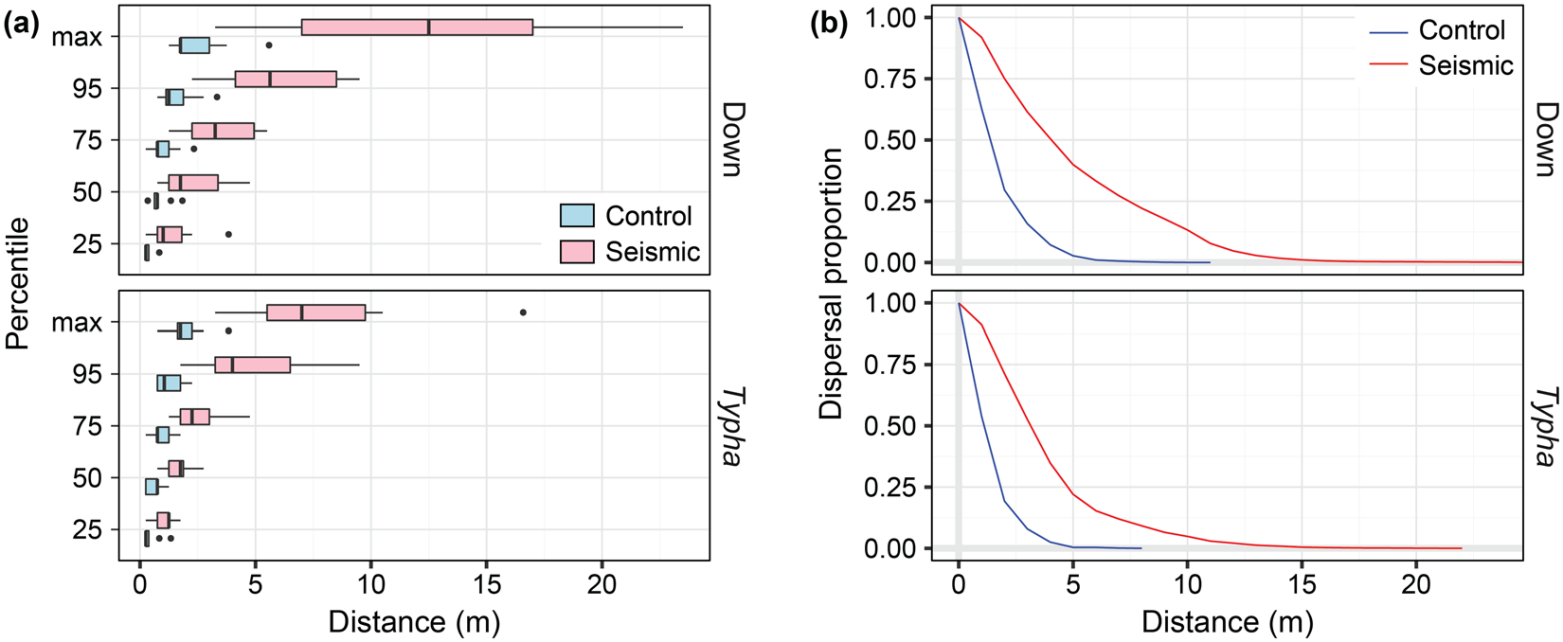
Dispersal patterns of experimental seed releases. (**a**) Dispersal distance percentiles and maxima for goose down (top) and *Typha latifolia* seed (bottom) for all plots (n = 12) in control forests (blue) and on seismic lines (red). Boxes represent interquartile ranges, whiskers extend to data ranges minus outliers (>1.5 interquartile range, shown as dots), and thick bars represent medians. (**b**) Dispersal attenuation curves showing the proportional dispersal of both goose down (top) and *Typha* seed (bottom) in control forest (blue) and on seismic lines (red).

Median dispersal distances for both testing materials on seismic lines (n = 24) compared with control forests (n = 24) were 291% farther (two-sample t-test: t = −6.68, df = 43.9, p = 3.37e-8), while 95^th^ percentile dispersal distances were 372% farther on seismic lines than in control forests (two-sample t-test: t = −9.51, df = 46.0, p = 1.98e-12) (Fig. 2b; Fig. 3). The 95^th^ percentile distances in the control forests were below the median distances on seismic lines for both seed types, indicating that over half the material along seismic lines is dispersing farther than nearly all the material in forested sites (Supplementary Table S1). While goose down (n = 24) consistently dispersed slightly farther than *Typha* (n = 24), the differences were not significant for median dispersal distance (t = 0.92, df = 45.7, p = 0.36) nor 95^th^ percentile dispersal distance (t = 0.93, df = 46.0, p = 0.36).

The substantial difference in both wind speeds and dispersal distances between control forests and seismic lines fits our expectation that higher wind speeds along forest dissections can increase seed dispersal distances. This relationship was confirmed with simple linear regressions of wind speed and median dispersal distance (n = 48, t = 5.07, r² = 0.36, p = 6.86e-6) and p95 dispersal distance (n = 48, t = 5.81, r² = 0.42, p = 5.59e-7).

Outputs from linear mixed models (LMMs) for median and 95^th^ percentile dispersal distances also support this conclusion: both show a significant effect on dispersal distances of categorical line width (control vs. narrow vs. wide), a variable that correlates highly (r = 0.81) with measured wind speed (Supplementary Table S2, Supplementary Fig. S1). Relative to control forests, the effect of narrow seismic lines, which had the highest mean wind speeds, was significant in the positive direction for median and 95^th^ percentile dispersal distances. Similarly, relative to control forests, the effect of wide seismic lines, which had slightly lower mean wind speeds than narrow lines, was also significant in the positive direction for both median and 95^th^ percentile dispersal distances. The categorical seed type (goose down vs. *Typha*) was only significant in the 95^th^ percentile distance model, but showed a similar trend of goose down dispersing farther than *Typha* for the median distance model. The only other variable in the final LMMs, the time interval between seed release and observation, was not significant in either the median or 95^th^ percentile distance models. The LMMs fit the data well, with the median and 95^th^ percentile models accounting for 70% and 79% of the total variance, respectively, with the fixed effects accounting for 53% and 71% of this variance, respectively. Complete LMM outputs are provided in Table 1 and the complete modelling process including model selection is provided in Supplementary Appendix 1.

**Table 1:** Linear mixed model results reporting on the factors affecting median and 95^th^ percentile dispersal distances.

Fixed effects	Median					95^th^ percentile				
	*β*	SE	df	t	p	*β*	SE	df	t	p
(Intercept)	−0.46	0.15	27.2	−3.11	4.33e-3	0.35	0.12	30.1	3.04	4.81e-3
Line width: control	0	—	—	—	—	0	—	—	—	—
Line width: wide	1.22	0.23	45.4	5.24	4.05e-6	1.49	0.19	46.4	7.74	6.89e-10
Line width: narrow	1.12	0.15	41.1	7.51	3.11e-9	1.28	0.12	41.1	10.37	4.87e-13
Seed type: down	0	—	—	—	—	0	—	—	—	—
Seed type: *Typha*	−0.22	0.13	35.6	−1.68	0.10	−0.22	0.11	35.3	−2.06	4.66e-2
Time interval	−0.25	0.38	19.4	−0.65	0.53	−0.30	0.30	18.3	−1.00	0.33
Time interval²	0.32	0.36	25.5	0.89	0.38	0.33	0.28	23.2	1.16	0.26
Random effects	*σ*²	*σ*	p_w_			*σ*²	*σ*	p_w_	*σ*²	
Site Number	0.11	0.33	0.96			0.06	0.24	0.92		
Residual	0.20	0.44				0.14	0.37			
Variance explained	r²_m_	r²_c_				r²_m_	r²_c_			
	0.53	0.70				0.71	0.79			

Predictors fitted in the final model as fixed effects were line width (3 classes: 1-control forest, 2-narrow seismic, 3-wide seismic), seed type (2 classes: 1-goose down, 2-*Typha latifolia*), and the time interval between release and observation (linear and quadratic). The site number (the unique location identifying the paired control and seismic transects) was included as a random effect. Variance explained by the fixed effects only (r²_m_) and by the entire model (r²_c_) are also reported.

Abbreviations: parameter estimate (*β*), standard error (SE), degrees of freedom (df), t-score (t), p-value (p), variance (*σ*²), standard deviation (*σ*), the p-value for the Shapiro-Wilk test fitted to test for the normality of random intercepts (p_w_, where p_w_ ‘>’ 0.05 indicates normality of random intercepts), the marginal r-squared (r²_m_) indicating variance explained only by fixed effects, and the conditional r-squared (r²_c_) indicating variance explained by the complete model.

### Loft testing

**Figure 4:**
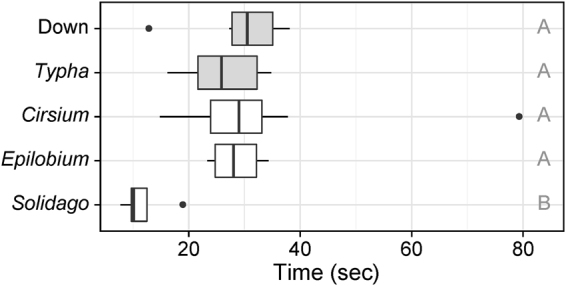
Loft testing results. The time (in seconds) required for the field release materials (in grey: goose down and *Typha latifolia*) and three other local species (*Cirsium arvense*, *Epilobium leptophyllum*, and *Solidago* spp.) to fall from a height of 6.6 m. Boxes represent interquartile ranges, whiskers extend to data ranges minus outliers (>1.5 interquartile range, shown as dots), and thick bars represent medians. Different letters indicate that groups are significantly different at p < 0.05 (Supplementary Table S3).

In the laboratory loft tests, *Solidago* spp. seed was the only tested seed or material that fell at a different rate, while all other materials fell at similar rates. In an ANOVA, the main effect of falling time was significant (F_4,41_ = 15.00, p = 2.64e-7). Tukey’s HSD comparisons indicated that, when dropped from a height of 6.6 m, the falling times for *Solidago* spp. were significantly different (shorter) than the other tested seeds or materials, including goose down (Δ means = −18.1 sec, p = 3.03e-5), *Typha* seed (Δ means = −15.2 sec, p = 1.35e-5), *Epilobium leptophyllum* seed (Δ means = −17.3 sec, p = 9.88e-6) and *Cirsium arvense* seed (Δ means = −21.8 sec, p = 1.02e-6). All other falling time comparisons were not statistically different at p < 0.05 (Fig. 4, Supplementary Table S3).

### Typical boreal forest wind conditions

**Figure 5:**
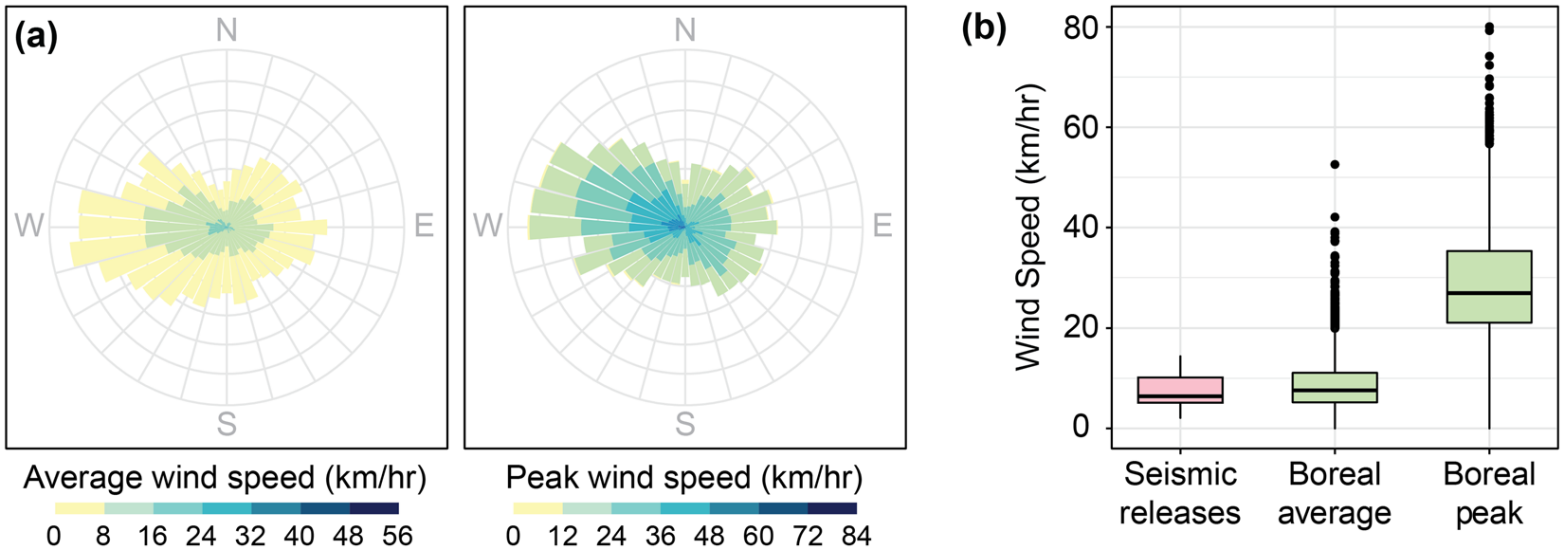
Regional wind patterns. (**a**) Frequencies of average (left) and peak (right) daily wind speeds and directions, for 115 weather stations throughout the Alberta boreal forest^48^, measured at 10 m above ground level through September and October of 2016 (the main seed dispersal season). Wind direction is reported as the direction from which the wind originates. (**b**) Comparison of wind speeds during the experimental seismic releases (measured at a height of 1.37 m) with the average and peak daily wind speeds during seed dispersal season, as measured at the Alberta boreal weather stations (measured at a height of 10 m). Boxes represent interquartile ranges, whiskers extend to data ranges minus outliers (>1.5 interquartile range, shown as dots), and thick bars represent medians.

Typical wind direction throughout the Alberta boreal forest during the season of seed dispersal, as measured at 115 Alberta boreal weather stations, was from the west and northwest for average and peak winds, respectively (Fig. 5a). Wind speeds measured during experimental releases along seismic lines at our test sites were similar to average wind speeds across the Alberta boreal forest, while peak wind speeds were notably lower than those recorded at boreal weather stations (Fig. 5b).

## Discussion

### Linear disturbances increase seed dispersal distance

Our results support our hypothesis that even narrow forest dissection can dramatically increase effective dispersal distances for wind-dispersed species, with median dispersal distances on seismic lines on average nearly three times larger than those in forested settings and 95^th^ percentile distances on average nearly four times larger. The dissections affected the dispersal curve by decreasing kurtosis and enhancing the positively skewed distribution that is typical of wind-dispersed seeds^27,29^. This trend supports previously published findings^14^ showing that anthropogenic linear disturbances generally increase dispersal. In our forested study area, narrow dissections are common and yet also quite expansive occurring over an area of 142,000 km^2^ (the extent of Alberta’s oil sands deposits). Effects in our tests were stronger at higher percentile distances, suggesting that forest dissections may have a greater effect on patterns of dispersal at longer distances.

Our results further support the conclusion that increased wind speed is largely responsible for the observed difference in dispersal. While not directly included in our final models, the strong positive linear relationship between wind and dispersal, the collinearity between wind speed and line width (the strongest explanatory variable in our LMM), as well as the strength of the wind speed effect in our alternative LMMs (Supplementary Appendix 2), all point to a strong positive effect of wind on dispersal. Further, the consistency of higher wind speeds along both line widths suggests a strong link between the presence of forest dissections and increased wind velocities, potentially via wind channelling. Interestingly, and while not significant, we found a consistent trend of slightly higher average dispersal along wide (7.5 to 8 m) lines while average wind speeds were slightly higher on narrow (5.3 to 6 m) lines. This may point to other mechanical processes that are limiting dispersal along narrow lines, such as seeds meeting forest edges sooner, thereby sheltering them from higher winds or due to different types of vegetation along seismic lines, such as tree stems or regenerating shrubs. While our experimental design was unable to separate the effect on dispersal of wind speed from that of such structural barriers, this would be a useful avenue of future investigation, particularly given the slow pace of natural regeneration along seismic lines^15,18^.

### Generalisability of results

The consistency in our loft tests for typical weedy species suggests that this trend is likely typical for many species that we did not test here. Further, our loft tests showing goose down falling ~11% slower than *Typha* and dispersing farther, are consistent with other studies suggesting that dispersal distances are related to seed falling velocities^30^. That said, the weaker effect of seed type in the models and the likely non-linear nature of this relationship makes it difficult to estimate from our data the direct effect of different falling speed on dispersal distance. A larger study with this specific focus would be informative.

Given the potential for wind speed to affect seed dispersal distance, it is also important to put our measured wind speeds in context of wind speeds observed on the surrounding landscape. While wind speeds observed in our trials were similar to the daily mean wind speeds for the larger boreal forest, they fell well short of the average daily peak wind speeds during the 2016 dispersal season, which were approximately double the highest speeds we observed on seismic lines during our tests. Given this disparity, the potential for increased seed dispersal along linear disturbances, particularly long-distance dispersal, through the course of a season may be underestimated in our field experiments suggesting potential effects of seismic lines to be even greater than reported here.

In our trials, it was not possible to quantify the proportion of seeds that were dispersed outside of the plotted transect with seed recovery often being lower than 100%. However, very few seeds were collected more than 15 m from the release point, implying that lost seeds were either dispersed in another direction, dispersed into the adjacent forest, or drawn upwards out of the seismic line by a wind uplift and therefore may have contributed to undetected long-distance dispersal.

### Invasive species vs. climate change adaptation

The North American boreal forest has long been viewed as hostile to introduced species, largely due to the inhospitable climate and relatively low biodiversity^31^. However, as has been shown in other regions, the fragmentation of otherwise intact forest is likely to increase the ability for all seed to spread more readily^32,33^. Seismic lines, due to their linear nature, may provide an ideal condition for such increased dispersal potential, particularly when strong winds are aligned with seismic line orientation. Such increased seed dispersal may pose a threat to native ecosystems through the increased movement of invasive species^31^.

On the other hand, increasing seed dispersal potential may also assist climate change adaptation for wind-dispersed species. This is recognised in the context of more stochastic long distance dispersal^34^, although consideration should also be given to increased short- and middle-distance dispersal, such as we observed in our disturbed sites. Even among common and widespread species, an increased ability to distribute seeds and pollen can allow species to adapt to locally changing environments through the movement of genetic material. While many native boreal species tend to be less sensitive to climatic environments and can tolerate a range of conditions (i.e. habitat generalists), invasive species also tend to exhibit high plasticity, making them effective colonisers^35^. There are also some notably rare habitat specialist in the Canadian boreal for which finding suitable habitats under future climates may be more challenging^36^. To be most successful, such native specialists would need to distribute systematically towards emerging suitable habitat in the future, whereas invasive species may benefit from distribution in non-ideal directions (i.e. that don’t track climate change as well).

In the boreal forest and elsewhere, future trends in extirpation of rare species or biotic homogenisation^37^ may depend on prevailing wind directions and their alignment with prolific linear disturbances. Given that we expect climate change to move boreal habitats northward and/or upslope^36,38^, prevailing winds and seismic lines could assist climate change adaptation if they are generally north-south oriented and have corresponding dominant southerly winds. However, in the Alberta boreal forest, where seismic lines are generally oriented on cardinal directions (east-west or north-south), prevailing winds during the main seed dispersal season are predominantly from the west, while peak winds tend to be predominantly from the northwest: largely in the opposite direction than would be beneficial for tracking suitable future climate habitats. That said, complex airflow patterns, such as wind channelling and turbulence, have been shown to affect both seed release and dispersal^39,40^ making specific conservation prescriptions in the context of linear developments uncertain.

In any region with a vast network of linear disturbances, connectivity planning should consider seed dispersal of both endemic and invasive species. Based on these results from our field experiments, the potential for narrow forest dissections associated with hydrocarbon exploration to promote seed dispersal and thus act as anthropogenic corridors for invasion is high and should be considered as an important consequence of resource development in the area. At least until the specifics of these dynamics are clarified, we advocate that conservation decisions incorporate not only connectivity, as they often do, but also specifically dissection connectivity within the context of local wind dynamics and, if there is particular concern with invasive species, restoration of seismic lines to forest vegetation.

## Methods

### Study area

Twelve seed release tests were performed in the late fall of 2015 near Ellscott, Alberta, Canada (54.5°N, 112.9°W), all at unique upland forest sites within an area of approximately 1 km^2^ (Fig. 1c). Late fall was chosen since this would be the period when many of the seeds from ‘weedy’ species are dispersed. Release sites consisted of a seismic line transect as well as a paired control forest transect ~20 m into the forest and parallel to the seismic line. Seismic line sites were selected for minimum regenerative cover to best represent recently-disturbed seismic lines where site conditions would be most similar to those conditions most suitable for colonization of invasive species. All transects were 25 m long with seismic lines measuring between 5.3 m and 8.0 m wide. Five release sites were on east-west oriented seismic lines, and seven were on north-south oriented seismic lines. Orientation of seismic line sites was based on the direction of prevailing winds at the time of the experiment in order to be approximately parallel to wind direction.

Dominant tree species in the study area was *Picea glauca* (white spruce) with occasional stands of *Pinus banksiana* (jack pine) and mixed areas of *Populus tremuloides* (trembling aspen). The study area was on public crown land with abundant evidence of recreational use, particularly off-highway vehicles and snowmobiles that help to maintain these features as open dissections. No significant bodies of water or topographic features were present in the study area.

### Seed releases

Tests were performed with seeds of *Typha latifolia*, an archetypal wind-dispersed plant species for which seeds are easily-obtainable and native to the areas wetlands but not uplands where experiments are occurring, and with commercial goose down as a proxy for other wind-dispersed species. These two seed-types were selected as they posed little to no threat of permanent colonisation on the site, thus minimising impacts of the research on the local plant community. *Typha* seeds and goose down were prepared using IFWB-C0 fluorescent clear blue dye, a commercial water tracer purchased from Risk Reactor Inc. (http://www.riskreactor.com). The dye was dehydrated, crushed to produce a powder, mixed with corn starch, and then scattered over the cattail seeds and down. We used a handheld UV LED lamp after nightfall to visually identify seeds post-dispersal when seeds where most visible (Supplementary Fig. S3).

We simultaneously released 1.0 g of *Typha* seed (several thousand seeds) and 2.5 g of goose down (~20 cm diameter ball of feathers) from a height of 1.37 m in the approximate centre of each seismic line. Release events were not timed for wind gusts, nor were testing days chosen based on forecast wind speed thus being representative of normal conditions. The precise number of seeds and down released was not recorded as results were considered based on proportional amounts of seed and down within sequential quadrats away from the release site. Release height (breast height) represents the approximate height of tall weedy herbaceous species such as native fireweed (*Chamaenerion angustifolium*) and non-native thistles (e.g. *Cirsium arvense*). Seed releases and post-dispersal counts were separated by a random time interval between 2 minutes and 16 hrs (mean = 6 hrs 22 min). Seeds were counted in the dominant dispersal direction out to 25 m at a 0.5 m increment for the first 5.0 m and then in 1.0 m increments for the remaining 20 m. On all seismic line tests, wind direction was measured to be within 45° of parallel to the seismic line, ensuring that dispersal was longitudinal to the disturbance rather than perpendicular into the adjacent forest. The top wind speed during the experimental release was measured using a Skywatch Meteos anemometer (http://meteo.jdc.ch/) at the same 1.37 m seed release height.

### Data analyses

All data analysis was performed in the R program for statistical computing^41^. The ANOVA and Tukey’s HSD tests for wind speeds on different line types, the t-test of dispersal distances on different line types, as well as the initial linear regression of dispersal distance and wind speeds, were performed using the base *stats* package for R. In all cases, wind speeds and dispersal distances were natural log transformed to improve normality of data and/or residuals.

In an effort to identify the key drivers of dispersal distances, we also fitted linear mixed models (LMMs) using the *lmer* command from the *lme4* and *lmerTest* packages for R^42^, using data from the 12 control and 12 seismic line tests for both *Typha* and goose down (n = 48). The *lmerTest* package for R^43^ was used to calculate p-values for LMMs, using Satterthwaite approximations to degrees of freedom for t-tests. Dispersal distance responses were natural log transformed before analysis to improve normality and homogeneity of residuals.

LMMs were fitted using either median or 95^th^ percentile dispersal distances as a response variable. The median distance was chosen to represent the dispersal distance of the bulk of seed material, while the 95^th^ percentile distance was used as an indication of immediate-area long-distance dispersal. The measured seismic line width was converted to a categorical variable to establish a control treatment (zero width in the adjacent control forest) as well as to address a strong bimodal distribution of different line widths: 5.3 to 6 m (narrow) and 7.5 to 8 m (wide).

We began by formulating a full model based on *a-priori* hypotheses, then performed a comprehensive simplification of our full model using both a manual stepwise AIC and a model dredging^44^ approach. Results from the full modelling process are provided in Supplementary Appendix 1 and we present only the results of our final model in the main text. Prior to model fitting, we assessed collinearity between explanatory variables using the *cor* and *pairs* command from the base *stats* package for R (Supplementary Table S2, Supplementary Fig. S1). Due to the high correlation between categorical line width and measured wind speed (r = 0.81), it was inappropriate to include both in the LMM. The categorical line width variable (our original treatment variable) was retained in the full model while wind speed was removed, as the former explained more variance in the models (p50 r² = 0.53 vs. 0.46; p95 r² = 0.71 vs. 0.63) and explicitly represented our experimental treatment, as well as producing models with lower AIC (p50 AIC = 88.2 vs. 99.2; p95 AIC = 68.7 vs. 85.2) (Supplementary Appendix 2).

In addition to categorical line width (control vs. narrow vs. wide), fixed effects in the full model included the categorical seed type (goose down vs. *Typha*), and the time interval (linear and quadratic) between seed release and observation (in hrs). The time interval was scaled using the *scale* command from the base *stats* package for R. The site number (the unique identifier of the paired control and seismic transects) was included as a random intercept. All interactions initially considered, including line width × seed type and line width × time interval, were removed from the model during simplification due to a lack of variable support (Supplementary Appendix 1). After simplification, the final LMM took the structure:

1 $${Dispersal}\,{Distance} \sim {Line}\,Width+{Seed}\,{Type}+{Time}+{Tim}{{e}}^{2}+({1}|{Site})$$

where *DispersalDistance* is the median or 95^th^ percentile dispersal distance, *LineWidth* is the seismic line width class (control vs. narrow vs. wide), *SeedType* is the seed type class (*Typha* vs. goose down), *Time* is the time interval in hours between release and observation, and *Site* is the unique site ID of each control and seismic test location.

Model effects were assessed and plotted with 95% marginal confidence intervals using the *effects* package for R^45^. Variance explained by the model, either by the entire model or by the fixed effects only (as conditional or marginal r², respectively), was calculated using *MuMIn* package for R^44,46^. Model assumptions of homogeneity of residuals (after log-transformation of the response variables), normality of residuals, and normality of the random intercepts were all met. Influence assessments were performed using the *influence.ME* package for R^47^. Plots of Cook’s distance did not identify any data exerting unreasonable influence over the models. While plots of standardised differences of the betas (dfbetas) suggested that certain data points may be disproportionately influential, removing these points from the final models did not change the interpretation of model outputs.

### Loft testing

We conducted laboratory loft tests to ensure that the loft of our dyed seed-types was representative of other weedy invasive species in the region. Specifically, our experimental materials, dyed commercial down and *Typha* seed, were compared against three locally invasive species: *Cirsium arvense* (Canada thistle), *Epilobium leptophyllum* (bog willow herb), and *Solidago* spp. (goldenrod). To estimate loft, ten individual seeds of each species were dropped from a 6.6 m high platform in a controlled, windless, indoor environment with fall times recorded to the nearest second. Falling times in loft tests were compared with a multi-level ANOVA and Tukey’s HSD test, both from the base *stats* package for R. Falling times were natural log transformed to improve normality of residuals.

### Local wind patterns

Wind speeds and directions measured on our test sites (forest and seismic line) were compared with general wind patterns in the region based on publicly available wind data from 115 weather stations scattered throughout Alberta’s boreal forest^48^ (Supplementary Fig. S3). We retrieved daily wind direction and intensity data for the months of September and October 2016, the period of most natural seed dispersal in the area. Average synoptic and peak wind speeds and directions were measured at 10 m above ground level (compared to 1.37 m high at experimental sites). Directional wind data were processed using the *circular* package for R^49^.

### Reproducibility

Recorded data from both the field site releases and the laboratory loft tests are provided as part of the Supplementary Information.

## Electronic supplementary material

Supplementary Information

Supporting Information

## References

[1] Fahrig L (2003). Effects of habitat fragmentation on biodiversity. Annu. Rev. Ecol. Evol. Syst..

[2] Scheffer M, Carpenter S, Foley JA, Folke C, Walker B (2001). Catastrophic shifts in ecosystems. Nature.

[3] Young A, Boyle T, Brown T (1996). The population genetic consequences of habitat fragmentation for plants. Trends Ecol. Evol..

[4] Sakai AK (2001). The population biology of invasive species. Annu. Rev. Ecol. Syst..

[5] Saunders DA, Hobbs RJ, Margules CR (1991). Biological consequences of ecosystem fragmentation: a review. Conserv. Biol..

[6] Resasco J (2014). Landscape corridors can increase invasion by an exotic species and reduce diversity of native species. Ecology.

[7] Jaeger JAG (2000). Landscape division, splitting index, and effective mesh size: new measures of landscape fragmentation. Landsc. Ecol..

[8] Trombulak SC, Frissell CA (2000). Review of ecological effects of roads on terrestrial and aquatic communities. Conserv. Biol..

[9] Coffin AW (2007). From roadkill to road ecology: a review of the ecological effects of roads. J. Transp. Geogr..

[10] Forman, R. T. T. *Road ecology: science and solutions* (Island Press, 2003).

[11] Nathan R, Muller-Landau HC (2000). Spatial patterns of seed dispersal, their determinants and consequences for recruitment. Trends Ecol. Evol..

[12] Dorp D, van Hoek WPMvanden, Daleboudt C (1996). Seed dispersal capacity of six perennial grassland species measured in a wind tunnel at varying wind speed and height. Can. J. Bot..

[13] Nathan R (2011). Spread of North American wind-dispersed trees in future environments. Ecol. Lett..

[14] Damschen EI (2014). How fragmentation and corridors affect wind dynamics and seed dispersal in open habitats. Proc. Natl. Acad. Sci..

[15] Lee P, Boutin S (2006). Persistence and developmental transition of wide seismic lines in the western Boreal Plains of Canada. J. Environ. Manage..

[16] Seitz NE, Westbrook CJ, Dubé MG, Squires AJ (2013). Assessing large spatial scale landscape change effects on water quality and quantity response in the lower Athabasca River basin. Integr. Environ. Assess. Manag..

[17] ABMI. Alberta Biodiversity Monitoring Institute, Wall-to-Wall Human Footprint Inventory, Digital Dataset. (2012). Available at: http://www.abmi.ca/home/data-analytics/da-top/da-product-overview/GIS-Human-Footprint-Land-Cover-Data/HF-inventory.html (Accessed: 1st March 2016).

[18] van Rensen CK, Nielsen SE, White B, Vinge T, Lieffers VJ (2015). Natural regeneration of forest vegetation on legacy seismic lines in boreal habitats in Alberta’s oil sands region. Biol. Conserv..

[19] Bayne EM, Wilgenburg SLV, Boutin S, Hobson KA (2005). Modeling and field-testing of Ovenbird (*Seiurus aurocapillus*) responses to boreal forest dissection by energy sector development at multiple spatial scales. Landsc. Ecol..

[20] Tigner J, Bayne EM, Boutin S (2014). Black bear use of seismic lines in northern Canada. J. Wildl. Manag..

[21] Dyer SJ, O’Neill JP, Wasel SM, Boutin S (2002). Quantifying barrier effects of roads and seismic lines on movements of female woodland caribou in northeastern Alberta. Can. J. Zool. Ott..

[22] Latham ADM, Latham MC, Boyce MS, Boutin S (2011). Movement responses by wolves to industrial linear features and their effect on woodland caribou in northeastern Alberta. Ecol. Appl..

[23] Dickie M, Serrouya R, DeMars C, Cranston J, Boutin S (2017). Evaluating functional recovery of habitat for threatened woodland caribou. Ecosphere.

[24] Kemper JT, Macdonald SE (2009). Directional change in upland tundra plant communities 20-30 years after seismic exploration in the Canadian low-arctic. J. Veg. Sci..

[25] Malcolm JR, Markham A, Neilson RP, Garaci M (2002). Estimated migration rates under scenarios of global climate change. J. Biogeogr..

[26] Roberts DR, Hamann A (2016). Climate refugia and migration requirements in complex landscapes. Ecography.

[27] Bacles CFE, Lowe AJ, Ennos RA (2006). Effective seed dispersal across a fragmented landscape. Science.

[28] Haeussler S, Bedford L, Leduc A, Bergeron Y, Kranabetter JM (2002). Silvicultural disturbance severity and plant communities of the southern Canadian boreal forest. Silva Fenn..

[29] Bullock JM, Clarke RT (2000). Long distance seed dispersal by wind: measuring and modelling the tail of the curve. Oecologia.

[30] Jongejans E, Telenius A (2001). Field experiments on seed dispersal by wind in ten umbelliferous species (*Apiaceae*). Plant Ecol..

[31] Sanderson LA, Mclaughlin JA, Antunes PM (2012). The last great forest: a review of the status of invasive species in the North American boreal forest. For. Lond.

[32] Farwig N, Böhning-Gaese K, Bleher B (2006). Enhanced seed dispersal of Prunus africana in fragmented and disturbed forests?. Oecologia.

[33] Levey DJ, Tewksbury JJ, Bolker BM (2008). Modelling long-distance seed dispersal in heterogeneous landscapes. J. Ecol..

[34] Trakhtenbrot A, Nathan R, Perry G, Richardson DM (2005). The importance of long-distance dispersal in biodiversity conservation. Divers. Distrib..

[35] van Tienderen PH (1997). Generalists, specialists, and the evolution of phenotypic plasticity in sympatric populations of distinct species. Evolution.

[36] Barber QE, Nielsen SE, Hamann A (2016). Assessing the vulnerability of rare plants using climate change velocity, habitat connectivity, and dispersal ability: a case study in Alberta, Canada. Reg. Environ. Change.

[37] McKinney ML, Lockwood JL (1999). Biotic homogenization: a few winners replacing many losers in the next mass extinction. Trends Ecol. Evol..

[38] Gray LK, Hamann A (2013). Tracking suitable habitat for tree populations under climate change in western North America. Clim. Change.

[39] Nathan R (2002). Mechanisms of long-distance dispersal of seeds by wind. Nature.

[40] Skarpaas O, Auhl R, Shea K (2006). Environmental variability and the initiation of dispersal: turbulence strongly increases seed release. Proc. R. Soc. Lond. B Biol. Sci..

[41] R Core Team. *R: A language and environment for statistical computing*. (R Foundation for Statistical Computing. http://www.R-project.org/, 2017).

[42] Bates D, Mächler M, Bolker B, Walker SC (2015). Fitting linear mixed-effects models using lme4. J. Stat. Softw..

[43] Kuznetsova, A., Brockhoff, P. B. & Christensen, R. H. B. *lmerTest: Tests in Linear Mixed Effects Models. R package version 2.0-33*. https://cran.r-project.org/web/packages/lmerTest (2016).

[44] Barton, K. *MuMIn: Multi-Model Inference, v1.13. 4*, http://CRAN.R-project.org/package=MuMIn (2013).

[45] Fox J (2003). Effect Displays in R for Generalised Linear Models. J. Stat. Softw..

[46] Nakagawa S, Schielzeth H (2013). A general and simple method for obtaining R^2^ from generalized linear mixed-effects models. Methods Ecol. Evol..

[47] Nieuwenhuis, R., Te Grotenhuis, M. & Pelzer, B. Influence.ME: tools for detecting influential data in mixed effects models. R J. 4, 38–47 (2012). *R J*. **4**, 38–47 (2012).

[48] Government of Alberta. Alberta Climate Information Service (ACIS). Ministry for Agriculture and Forestry, available http://agriculture.alberta.ca/acis/ (2016).

[49] Agostinelli, C. & Lund, U. *Circular: circular statistics. R package version 0.4-7*. https://r-forge.r-project.org/projects/circular/ (2013).

[50] Brandt JP (2009). The extent of the North American boreal zone. Environ. Rev..

