# Supplementary material for: Accelerated seed dispersal along linear disturbances in the Canadian oil sands region
Source: Sci Rep. 2018 Mar 19;8:4828. doi: 10.1038/s41598-018-22678-y (PMC5859175; doi:10.1038/s41598-018-22678-y)
Supplement: Supplementary file 1 — Supplementary Information [file 41598_2018_22678_MOESM1_ESM.pdf]

Accelerated seed dispersal along linear disturbances in the Canadian oil sands region

David Roberts [1, 2], **Simone Ciuti** [3], **Quinn Barber** [4], **Caitlin Willier** [4], **Scott E. Nielsen** [4]

[1] Department of Geography, University of Calgary, ES-356, 2500 University Dr. NW, Calgary, Alberta, T2N 1N4, Canada

[2] Arctic Institute of North America, University of Calgary, ES-1040, 2500 University Dr. NW, Calgary, Alberta, T2N 1N4, Canada

[3] School of Biology and Environmental Science, University College Dublin, Science West, Belfield, D4, Dublin, Ireland

[4] Department of Renewable Resources, University of Alberta, 705 General Services Building, Edmonton, Alberta, T6G 2H1, Canada

Supplementary Tables

Table S1: Dispersal distance percentiles 1

Table S2: Correlation coefficients of the explanatory variables 2

Table S3: Results of the loft testing 3

Supplementary Figures

Figure S1: Correlation plot of the explanatory variables 4

Figure S2: Photos of the seed release setup in the field 5

Table S3: Locations of weather stations throughout the Alberta boreal forest 6

Appendices

Appendix 1: Linear mixed model selection and outputs 7

Appendix 2: Linear regression of wind speed and dispersal 26

Supplementary Tables

Table S1: Dispersal distance percentiles (25th, 50th, 75th, and 95th) and maxima (Max) in metres, averaged across all plots (n = 12, with standard errors in brackets) for goose down and *Typha latifolia* seed in control forests or on seismic lines.

	25th	50th	75th	95th	Max
Goose down					
Control	0.38 (0.07)	0.75 (0.12)	1.04 (0.12)	1.58 (0.16)	2.52 (0.36)
Seismic	1.40 (0.28)	2.33 (0.39)	3.52 (0.39)	5.92 (0.46)	12.92 (2.07)
Typha					
Control	0.42 (0.10)	0.63 (0.11)	0.92 (0.11)	1.28 (0.14)	1.92 (0.23)
Seismic	1.08 (0.13)	1.67 (0.16)	2.50 (0.16)	4.74(0.31)	8.38 (1.28)

Table S2: Correlation coefficients (Pearson's r) of the explanatory variables considered in our study. Note that seed type and line width are categorical variables, upon which the correlation with other predictors depends on the order of categories. The order of seed type (goose down vs. *Typha latifolia*) is inconsequential as it contains only two levels. Here, we have ordered the line width categories (1 = control, 2 = wide, 3 = narrow) in order of increasing wind speed (**Figure 2a**) to determine the maximum potential covariance for model variable selection. Note the high collinearity (r = 0.81) between wind speed and line width. A correlation plot is also provided in **Figure S2**.

	Seed type	Line width	Time interval	Wind speed
Seed type	1.00			
Line width	0.00	1.00		
Time interval	0.00	-0.19	1.00	
Wind speed	0.00	0.81	-0.22	1.00

Table S3: (a) Results of the loft testing, showing the average time (Time, in seconds with standard errors in brackets) required for seeds of each species or goose down to fall from a height of 6.6 m. The difference between each species and the *Typha latifolia* or goose down tests is also shown, both in seconds (Δ Down and Δ *Typha*) and by proportion (α Down and α *Typha*). **(b)** Comparisons of the loft testing data from a one-way analysis of variance (ANOVA) using a Tukey HSD test for multiple comparisons, showing the difference in means (Δ mean), the 95% lower (lwr) and upper (upr) confidence intervals, and the adjusted p-value (p-adj). Note that falling times were natural log transformed prior to the ANOVA to improve normality of residuals.

(a)

Material	Time	Δ Down	Δ *Typha*	α Down	α *Typha*
Goose down	29.2 (3.7)	0.0	2.9	1.00	1.11
Typha latifolia	26.3 (2.1)	-2.9	0.0	0.90	1.00
Chamerion angustifolium	28.5 (1.7)	-0.7	2.2	0.97	1.08
Circium arvense	32.9 (6.2)	3.7	6.6	1.13	1.25
Solidago spp.	11.2 (1.1)	-18.1	-15.2	0.38	0.42

(b)

Species comparison	Δ mean	lwr	upr	p-adj
Experimental materials				
Down-*Typha*	0.08	-0.40	0.56	0.988
Goose down				
Down-*Cirsium*	-0.07	-0.56	0.42	0.994
Down-*Epilobium*	-0.02	-0.54	0.50	1.000
Down-*Solidago*	0.94	0.45	1.43	3.03e-5
Typha seed				
Typha-Cirsium	-0.15	-0.58	0.28	0.846
Typha-Epilobium	-0.10	-0.56	0.36	0.970
Typha-Solidago	0.86	0.43	1.29	1.35e-5
Other materials				
Epilobium-Cirsium	-0.05	-0.52	0.42	0.998
Solidago-Cirsium	-1.01	-1.45	-0.57	1.02e-6
Solidago-Epilobium	-0.96	-1.43	-0.49	9.88e-6

Supplementary Figures

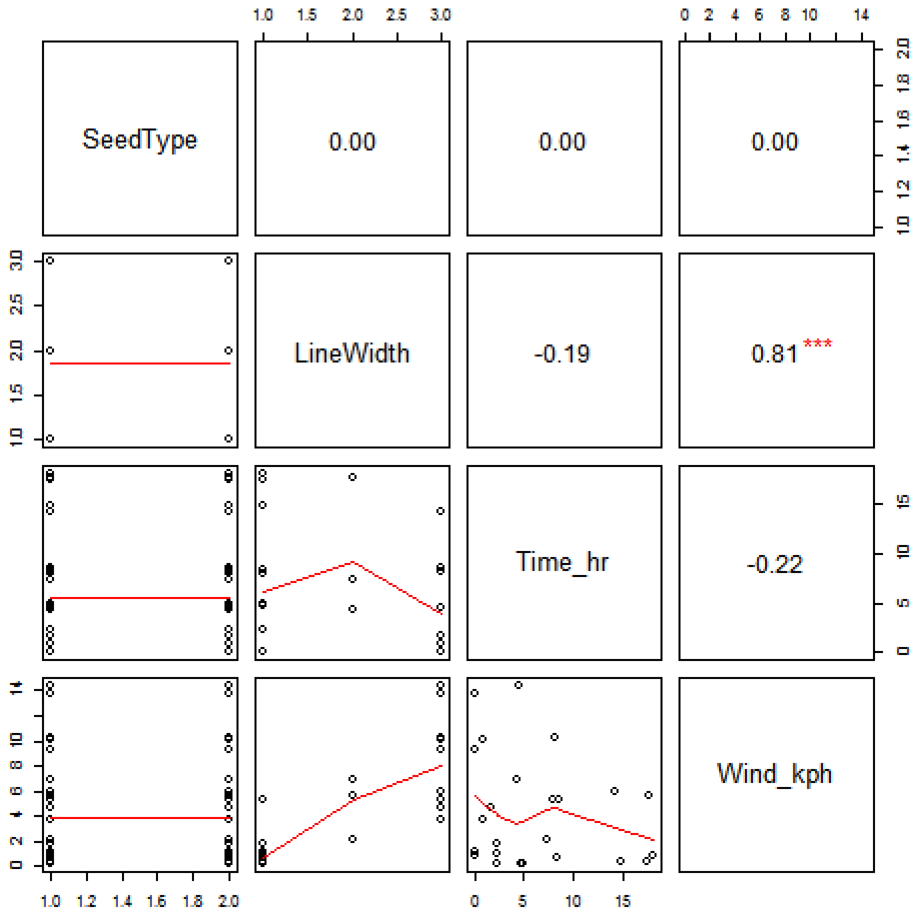

Figure S1: Correlation plot of the explanatory variables considered in our study. Note that seed type (SeedType) and line width (LineWidth) are categorical variables, upon which the correlation with other predictors depends on the order of categories. The order of seed type (goose down vs. *Typha latifolia*) is inconsequential as it contains only two levels. Here, we have ordered the line width categories (1 = control, 2 = wide, 3 = narrow) in order of increasing wind speed (**Figure 3a**) to determine the maximum potential covariance for model variable selection. Significance codes: *** $p < 0.001$; ** $p < 0.01$; * $p < 0.05$. A table of correlations is also provided in **Table S2**.

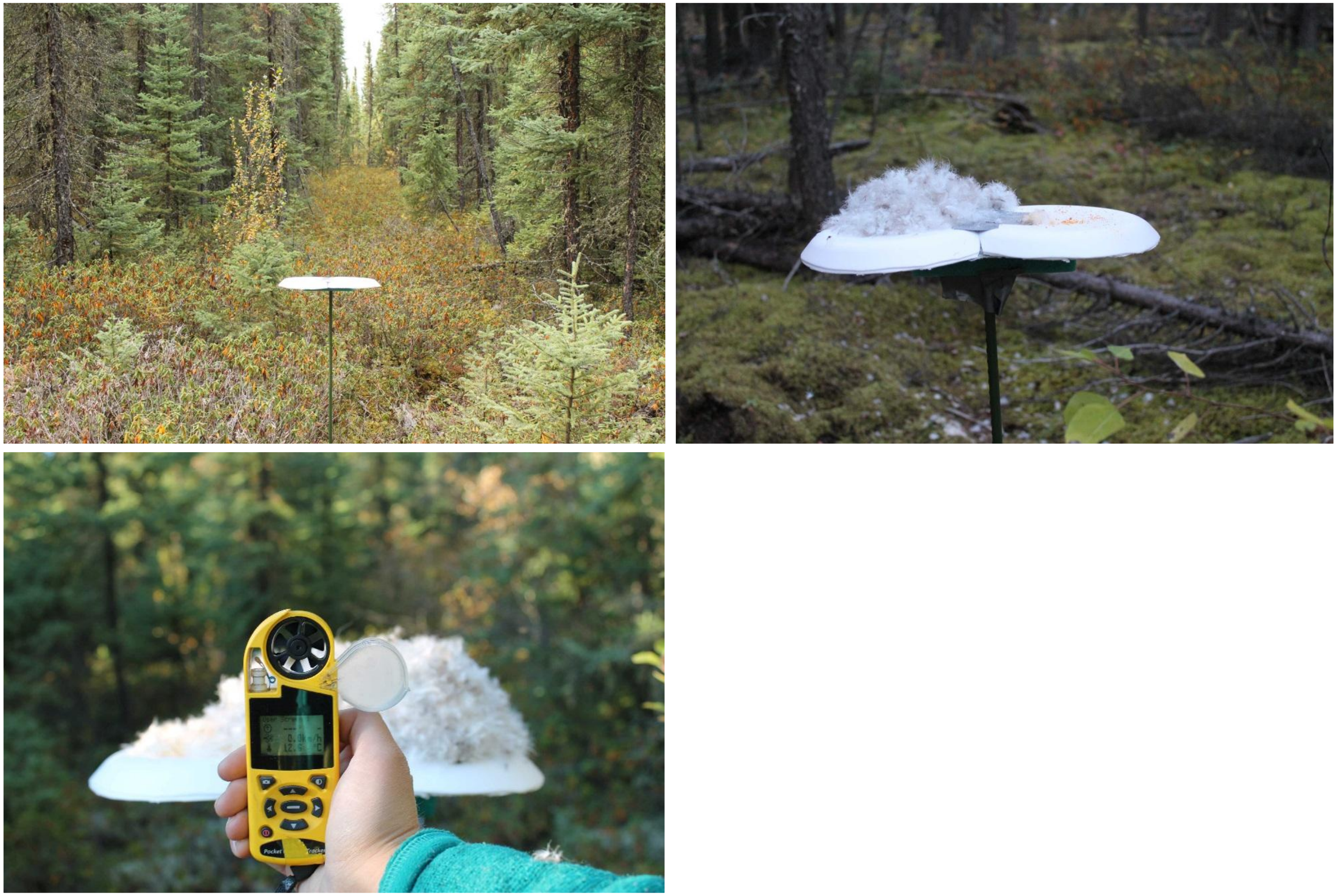

Figure S2: Photos of the seed release setup in the field. **(Top left)** the release platform on a seismic line, **(top right)** *Typha latifolia* seed goose down prepared for release on forested site, **(bottom)** goose down and *Typha latifolia* seed during release (note that different anemometers were used in the field during actual experimental trials, see Methods).

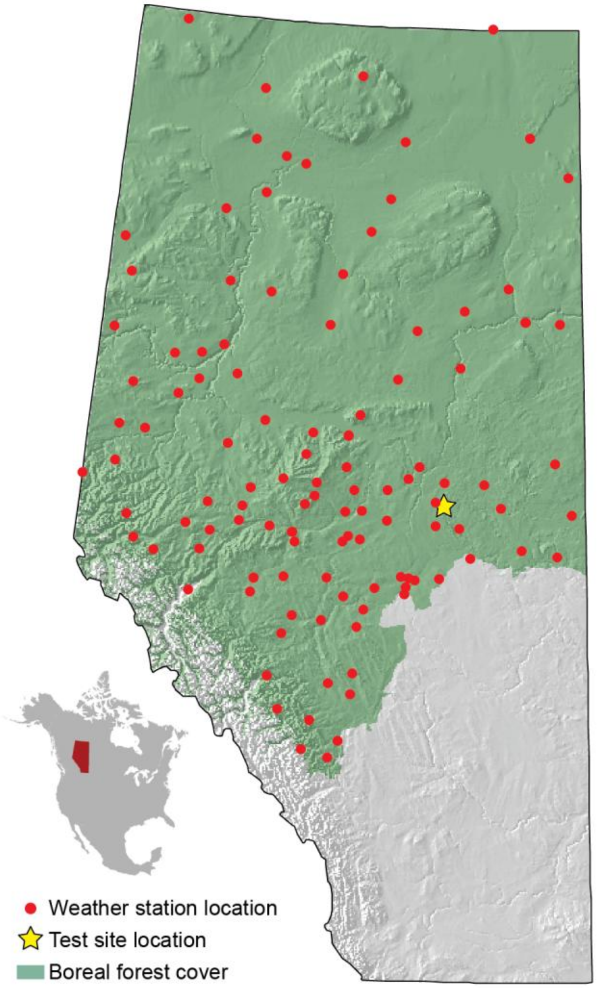

Figure S3: Locations of weather stations throughout the Alberta boreal forest used to summarise prevailing wind speeds and directions. Weather station locations are shown as red dots and the Alberta boreal forest extent is shown in green[1]. Weather station data was provided by the Ministry for Agriculture and Forestry of the Government of Alberta, Alberta Climate Information Service (ACIS), available at http://agriculture.alberta.ca/acis/. Map created in ESRI ArcMap v10.4 (http://www.esri.com/).

Appendix 1: Linear mixed modelling (LMM)

Modelling approach overview

We performed linear mixed modelling (LMM) to determine contributing factors to median (p50) and 95th percentile (p95) seed dispersal distances. This was a five step process of model selection and final model fitting. It involves two model selection approaches, manual stepwise selection[2] and model dredging[3,4]. We note that this approach is not driven by a lack of thinking about the problem of interest: our full model (Step 1) has been defined based on clear *a-priori* hypotheses (see main text of the paper as well as below for our rationale in including single terms and some of their interactions). Rather, our goal is to make sure that alternative model selection approaches, used to reduce the complexity of our starting *a-priori* model, lead to the same conclusion, thus increasing confidence in our results for readers preferring one approach over another[5].

Briefly, the process involves:

Step 1 Fit the full model, including all variables and interactions identified in *a-priori* hypotheses.

Step 2 Perform first model selection routine using a manual backward stepwise variable selection.

Step 3 Dredge the full model to identify the best models based on AIC.

Step 4 Choose a final model structure based on the stepwise and dredging approaches.

Step 5 Fit the final model.

In the preliminary full model, explanatory variables (fixed effects) include:

LineWidth Categorical (3 levels), differentiating between (1) forested control sites, (2) wide seismic lines, and (3) narrow seismic lines. Note that categories were ordered such that they identify the maximum correlation with measured wind speed, which we now drop from the models due to high covariance (Table S3, Figure S3).

SeedType Categorical (2 levels), differentiating between (1) goose down, and (2) *Typha latifolia* seed.

Time_hr Time (in hours) between experimental seed release and seed observation and collection. We consider the linear and quadratic form of this variable.

We consider the following interactions in the full model:

LineWidth × Time_hr Testing whether the effect of the line width (collinear with wind speed) is consistent across observation time intervals.

LineWidth × SeedType Testing whether the effect of line width (collinear with wind speed) is consistent for both goose down and *Typha latifolia* seed.

Finally, we include as a random effect in all models:

Site The unique site number of each paired control and seismic test location

The resulting full model took the structure:

$$\textit{Dispersal Distance} \sim \textit{Line Width} + \textit{Seed Type} + \textit{Time} + \textit{Time}^2 + \textit{Line Width} \times \textit{Time} + \textit{Line Width} \times \textit{Seed Type} + (1|\textit{Site})$$

Models were fitted using the *lmer* command from the *lme4* package for R[6]. Estimate p-values were added with the *lmerTest* package for R[7]. Plots of model effects were created with the *effects* package for R[7], with shaded areas showing 95% marginal confidence intervals. Dredging, dredged model averaging, and calculation of variance explained by the mixed models are performed with the *MuMIn* package for R[3]. Note that the *MuMIn* package provides two measures of variance explained: Marginal R_GLMM^2 represents the variance explained by fixed effects only while Conditional R_GLMM^2 represents the variance explained by then entire model (combined fixed and random effects).[3]

The same model selection procedures were carried out separately for p50 and for p95 distances as a response variable. Results of both are presented below in Steps 1-3. The selection procedure identified very similar model structures for both p50 and p95. For this reason, as well as to maintain comparable model outputs, we select a single final model structure (Step 4) that is comprehensive for both p50 and p95.

Model selection for 50th percentile dispersal distances (p50)

Step 1 – Fit the full model

```
Formula: log(p50) ~ LineWidth_cat + SeedType + scale(Time_hr) + scale(Time_hr^2) +
    LineWidth_cat * scale(Time_hr) + SeedType * LineWidth_cat + (1 | Site_No)
   Data: moddat

     AIC      BIC   logLik deviance df.resid
    93.8    116.3    -34.9     69.8       36

Scaled residuals:
     Min       1Q   Median       3Q      Max
-2.35601 -0.58693 -0.08495  0.81367  1.52555

Random effects:
 Groups   Name        Variance Std.Dev.
 Site_No  (Intercept) 0.1274   0.3570
 Residual             0.1791   0.4233
Number of obs: 48, groups:  Site_No, 12

Fixed effects:
                                   Estimate Std. Error      df t value Pr(>|t|)
(Intercept)                         -0.4555     0.1604 29.5800  -2.840  0.00809 **
LineWidth_catWide                    0.9825     0.3259 40.8600   3.015  0.00440 **
LineWidth_catNarrow                  1.1609     0.2017 38.9900   5.756 1.13e-06 ***
SeedTypeTypha                       -0.2111     0.1728 34.3100  -1.222  0.23006
scale(Time_hr)                      -0.3733     0.4593 22.3400  -0.813  0.42492
scale(Time_hr^2)                     0.3990     0.4230 27.5500   0.943  0.35372
LineWidth_catWide:scale(Time_hr)     0.3360     0.2346 43.1900   1.432  0.15925
LineWidth_catNarrow:scale(Time_hr)   0.1090     0.1892 42.5300   0.576  0.56743
LineWidth_catWide:SeedTypeTypha      0.1169     0.3864 34.3100   0.303  0.76395
LineWidth_catNarrow:SeedTypeTypha   -0.0499     0.2640 34.3100  -0.189  0.85117
---
Signif. codes:  0 '***' 0.001 '**' 0.01 '*' 0.05 '.' 0.1 ' ' 1

      R2m       R2c
0.5457026 0.7345497
```

Step 2 – Backward stepwise variable selection

Individual explanatory variables were removed from the full model one at a time, then the model was re-run. The variable with the smallest effect on the response (i.e. the highest p-value) was removed at each iteration. Model selection was considered complete when all remaining variables were significant at $p < 0.05$. For each iteration (Run), we report the AIC, the variance explained by the fixed effects (r^2_m), and the variables with the highest p-value in the model output (Highest p), with the p-value in brackets.

Run	Model Description	AIC	r^2_m	Highest p
0	Full model	93.8	0.55	LineWidthNarrow:SeedTypeTypha (0.85)
1	Without LineWidth × SeedType	90.0	0.54	LineWidthNarrow:Time_hr (0.57)
2	Without LineWidth × Time	88.2	0.53	Time_hr (0.53)
3	Without linear Time	86.6	0.53	Time_hr^2 (0.37)
4	Without quadratic Time	85.5	0.52	SeedTypeTypha (0.11)
5	Without SeedType	86.1	0.50	LineWidthWide (7.36e-6)

The backward stepwise AIC results in the following model:

```
Formula: log(p50) ~ LineWidth + (1 | Site_No)
   Data: moddat

     AIC      BIC   logLik deviance df.resid
    86.1     95.4    -38.0     76.1       43

Scaled residuals:
     Min       1Q   Median       3Q      Max
-2.01580 -0.66166 -0.00707  0.72576  1.71225

Random effects:
 Groups   Name        Variance Std.Dev.
 Site_No  (Intercept) 0.1009   0.3177
 Residual             0.2202   0.4692
Number of obs: 48, groups:  Site_No, 12

Fixed effects:
                Estimate Std. Error      df t value Pr(>|t|)
(Intercept)      -0.5547     0.1326 21.0700  -4.183 0.000417 ***
LineWidthWide     1.2251     0.2430 46.7700   5.042 7.36e-06 ***
LineWidthNarrow   1.0860     0.1512 39.6600   7.182 1.10e-08 ***
---
Signif. codes:  0 '***' 0.001 '**' 0.01 '*' 0.05 '.' 0.1 ' ' 1
```

Step 3 – Model selection by dredging

First, we dredge the full model:

```
Global model call: lme4::lmer(formula = log(p50) ~ LineWidth + SeedType + scale(Time_hr) +
    scale(Time_hr^2) + LineWidth * scale(Time_hr) + SeedType *
    LineWidth + (1 | Site_No), data = moddat, REML = F, na.action = "na.fail")
---
Model selection table
       (Int) LnW scl(Tim_hr) scl(Tim_hr^2) SdT LnW:scl(Tim_hr) LnW:SdT df  logLik  AICc delta weight
2  -0.554700   +                                                        5 -38.036  87.5  0.00  0.258
10 -0.447100   +                             +                          6 -36.727  87.5  0.00  0.258
6  -0.565500   +                  0.095400                              6 -37.674  89.4  1.90  0.100
14 -0.458400   +                  0.099890   +                          7 -36.325  89.4  1.95  0.097
4  -0.562300   +     0.07270                                            6 -37.842  89.7  2.23  0.085
12 -0.455100   +     0.07667                 +                          7 -36.510  89.8  2.32  0.081
8  -0.565300   +    -0.24080      0.314800                              7 -37.480  91.8  4.26  0.031
16 -0.458000   +    -0.24710      0.323900   +                          8 -36.117  91.9  4.42  0.028
42 -0.449200   +                             +                       +  8 -36.650  93.0  5.49  0.017
20 -0.558100   +     0.03223                                 +          8 -36.838  93.4  5.87  0.014
28 -0.450900   +     0.03681                 +               +          9 -35.421  93.6  6.08  0.012
46 -0.460500   +                  0.100200   +                       +  9 -36.245  95.2  7.73  0.005
44 -0.457200   +     0.07691                 +                       +  9 -36.431  95.6  8.10  0.004
24 -0.560400   +    -0.34860      0.371000                   +          9 -36.487  95.7  8.21  0.004
32 -0.453400   +    -0.37190      0.397400   +               +         10 -35.009  96.0  8.46  0.004
48 -0.460100   +    -0.24750      0.324500   +                       + 10 -36.036  98.0 10.52  0.001
60 -0.453000   +     0.03709                 +               +       + 11 -35.337 100.0 12.51  0.000
64 -0.455500   +    -0.37330      0.399000   +               +       + 12 -34.922 102.8 15.26  0.000
1   0.005668                                                            3 -57.310 121.2 33.66  0.000
9   0.113300                                 +                          4 -56.869 122.7 35.17  0.000
3   0.005668        -0.01216                                            4 -57.304 123.5 36.04  0.000
5   0.005668                     -0.004572                              4 -57.309 123.5 36.05  0.000
11  0.113300        -0.01291                 +                          5 -56.863 125.2 37.65  0.000
13  0.113300                     -0.005096   +                          5 -56.868 125.2 37.66  0.000
7   0.005668        -0.10100      0.092510                              5 -57.280 126.0 38.49  0.000
15  0.113300        -0.10210      0.092800   +                          6 -56.839 127.7 40.22  0.000
Models ranked by AICc(x)
Random terms (all models):
‘1 | Site_No’
```

We then use a cutoff of delta AIC < 4 to select the top models based on AIC[8,9]. These models can be averaged, providing averaged parameter estimates, weighted by AIC.

```
Call: model.avg(object = dredged[dredged$delta < 4, ])

Component model call:
::(formula = log(p50) ~ <6 unique rhs>, data = moddat, REML = F, na.action = na.fail)
     lme4(formula = log(p50) ~ <6 unique rhs>, data = moddat, REML = F, na.action = na.fail)
     lmer(formula = log(p50) ~ <6 unique rhs>, data = moddat, REML = F, na.action = na.fail)

Component models:
    df logLik  AICc delta weight
1    5 -38.04 87.50  0.00   0.29
14   6 -36.73 87.50  0.00   0.29
13   6 -37.67 89.40  1.90   0.11
134  7 -36.32 89.45  1.95   0.11
12   6 -37.84 89.73  2.23   0.10
124  7 -36.51 89.82  2.32   0.09
```

```
Term codes:
       LineWidth   scale(Time_hr) scale(Time_hr^2)         SeedType
               1                2                3                4

Model-averaged coefficients:
(full average)
                 Estimate Std. Error Adjusted SE z value Pr(>|z|)
(Intercept)      -0.50526    0.15024     0.15413   3.278  0.00104 **
LineWidthWide     1.21841    0.23905     0.24611   4.951    7e-07 ***
LineWidthNarrow   1.09877    0.15089     0.15531   7.075  < 2e-16 ***
SeedTypeTypha    -0.10682    0.14128     0.14307   0.747  0.45528
scale(Time_hr^2)  0.02193    0.06625     0.06750   0.325  0.74522
scale(Time_hr)    0.01406    0.05798     0.05928   0.237  0.81255

(conditional average)
                 Estimate Std. Error Adjusted SE z value Pr(>|z|)
(Intercept)      -0.50526    0.15024     0.15413   3.278  0.00104 **
LineWidthWide     1.21841    0.23905     0.24611   4.951    7e-07 ***
LineWidthNarrow   1.09877    0.15089     0.15531   7.075  < 2e-16 ***
SeedTypeTypha    -0.21523    0.12993     0.13382   1.608  0.10775
scale(Time_hr^2)  0.09761    0.11022     0.11353   0.860  0.38989
scale(Time_hr)    0.07464    0.11544     0.11890   0.628  0.53016
---
Signif. codes:  0 '***' 0.001 '**' 0.01 '*' 0.05 '.' 0.1 ' ' 1

Relative variable importance:
                     LineWidth SeedType scale(Time_hr^2) scale(Time_hr)
Importance:          1.00      0.50     0.22             0.19
N containing models:    6         3        2                2
```

Model selection for 95th percentile dispersal distances (p95)

Step 1 – Fit the full model

```
Formula: log(p95) ~ LineWidth + SeedType + scale(Time_hr) + scale(Time_hr^2) +
    LineWidth * scale(Time_hr) + SeedType * LineWidth + (1 | Site_No)
   Data: moddat

     AIC      BIC   logLik deviance df.resid
    74.1     96.6    -25.1     50.1       36

Scaled residuals:
    Min      1Q  Median      3Q     Max
-1.7234 -0.8084  0.1399  0.5974  2.2914

Random effects:
 Groups   Name        Variance Std.Dev.
 Site_No  (Intercept) 0.07164  0.2677
 Residual             0.12324  0.3511
Number of obs: 48, groups:  Site_No, 12

Fixed effects:
                               Estimate Std. Error       df t value Pr(>|t|)
(Intercept)                     0.34370    0.12786 31.58000   2.688   0.0114 *
LineWidthWide                   1.34839    0.26853 41.07000   5.021 1.04e-05 ***
LineWidthNarrow                 1.34769    0.16653 38.64000   8.093 7.62e-10 ***
SeedTypeTypha                  -0.19437    0.14332 33.68000  -1.356   0.1841
scale(Time_hr)                 -0.44655    0.36269 21.48000  -1.231   0.2316
scale(Time_hr^2)                0.42958    0.33593 26.00000   1.279   0.2123
LineWidthWide:scale(Time_hr)    0.26369    0.19282 43.46000   1.368   0.1785
LineWidthNarrow:scale(Time_hr)  0.14731    0.15566 42.61000   0.946   0.3493
LineWidthWide:SeedTypeTypha    -0.02155    0.32046 33.68000  -0.067   0.9468
LineWidthNarrow:SeedTypeTypha  -0.06355    0.21892 33.68000  -0.290   0.7734
---
Signif. codes:  0 '***' 0.001 '**' 0.01 '*' 0.05 '.' 0.1 ' ' 1

      R2m       R2c
0.7126228 0.8182708
```

Step 2 – Backward stepwise variable selection

Individual explanatory variables were removed from the full model one at a time and the model re-run. The variable with the smallest effect on the response (i.e. the highest p-value) was removed at each iteration. Model selection was considered complete when all remaining variables were significant at $p < 0.05$. For each iteration (Run), we report the AIC, the variance explained by the fixed effects (r^2_m), and the variables with the highest p-value in the model output (Highest p), with the p-value in brackets.

Run	Model Description	AIC	r^2_m	Highest p
0	Full model	74.1	0.71	LineWidthNarrow:SeedTypeTypha (0.95)
1	Without LineWidth × SeedType	70.2	0.71	LineWidthNarrow:Time_hr (0.35)
2	Without LineWidth × Time	68.7	0.71	Time_hr (0.33)
3	Without linear Time	67.7	0.70	Time_hr^2 (0.49)
4	Without quadratic Time	66.2	0.70	SeedTypeTypha (0.049)

The backward stepwise AIC results in the following model:

```
Formula: log(p95) ~ LineWidth + SeedType + (1 | Site_No)
   Data: moddat

     AIC      BIC   logLik deviance df.resid
    66.2     77.4    -27.1     54.2       42

Scaled residuals:
     Min       1Q   Median       3Q      Max
-1.84186 -0.81021  0.07474  0.74251  2.13361

Random effects:
 Groups   Name        Variance Std.Dev.
 Site_No  (Intercept) 0.05969  0.2443
 Residual             0.14134  0.3759
Number of obs: 48, groups:  Site_No, 12

Fixed effects:
                Estimate Std. Error      df t value Pr(>|t|)
(Intercept)       0.3591     0.1175 30.4500   3.056  0.00464 **
LineWidthWide     1.4779     0.1937 46.9400   7.628 9.25e-10 ***
LineWidthNarrow   1.2653     0.1210 39.5500  10.457 6.00e-13 ***
SeedTypeTypha    -0.2209     0.1085 35.7100  -2.035  0.04929 *
---
Signif. codes:  0 '***' 0.001 '**' 0.01 '*' 0.05 '.' 0.1 ' ' 1
```

Step 3 – Model selection by dredging

First, we dredge the full model:

```
Global model call: lme4::lmer(formula = log(p95) ~ LineWidth + SeedType + scale(Time_hr) +
    scale(Time_hr^2) + LineWidth * scale(Time_hr) + SeedType *
    LineWidth + (1 | Site_No), data = moddat, REML = F, na.action = "na.fail")
---
Model selection table
    (Int) LnW scl(Tim_hr) scl(Tim_hr^2) SdT LnW:scl(Tim_hr) LnW:SdT df  logLik  AICc delta weight
10 0.3591   +                             +                         6 -27.086  68.2  0.00  0.354
2  0.2486   +                                                       5 -29.046  69.5  1.30  0.185
14 0.3521   +                   0.06160   +                         7 -26.846  70.5  2.27  0.114
12 0.3556   +    0.033820                 +                         7 -27.020  70.8  2.62  0.096
6  0.2423   +                   0.05584                             6 -28.852  71.8  3.53  0.061
4  0.2457   +    0.028050                                           6 -29.000  72.0  3.83  0.052
16 0.3527   +   -0.296000       0.32920   +                         8 -26.362  72.4  4.20  0.043
8  0.2426   +   -0.297900       0.32710                             7 -28.370  73.5  5.32  0.025
42 0.3458   +                             +                       + 8 -27.049  73.8  5.57  0.022
28 0.3597   +   -0.006173                 +               +         9 -25.889  74.5  6.29  0.015
20 0.2499   +   -0.012120                                 +         8 -27.982  75.7  7.44  0.009
32 0.3570   +   -0.446100       0.42900   +               +        10 -25.113  76.2  7.95  0.007
46 0.3389   +                   0.06171   +                       + 9 -26.808  76.4  8.13  0.006
44 0.3423   +    0.033930                 +                       + 9 -26.983  76.7  8.48  0.005
24 0.2472   +   -0.420600       0.39940                   +         9 -27.331  77.4  9.18  0.004
48 0.3394   +   -0.296000       0.32930   +                       + 10 -26.324  78.6 10.37  0.002
60 0.3465   +   -0.006050                 +               +       + 11 -25.849  81.0 12.81  0.001
64 0.3437   +   -0.446500       0.42960   +               +       + 12 -25.071  83.1 14.84  0.000
1  0.9079                                                           3 -57.982 122.5 54.29  0.000
9  1.0180                                 +                         4 -57.531 124.0 55.77  0.000
3  0.9079       -0.047560                                           4 -57.900 124.7 56.51  0.000
5  0.9079                      -0.03260                             4 -57.944 124.8 56.60  0.000
11 1.0180       -0.047560                 +                         5 -57.448 126.3 58.10  0.000
13 1.0180                      -0.03260   +                         5 -57.492 126.4 58.19  0.000
7  0.9079       -0.211100       0.17020                             5 -57.820 127.1 58.85  0.000
15 1.0180       -0.211100       0.17020   +                         6 -57.366 128.8 60.56  0.000
Models ranked by AICc(x)
Random terms (all models):
‘1 | Site_No’
```

We then use a cutoff of delta AIC < 4 to select the top models based on AIC. These models can be averaged, providing averaged parameter estimates, weighted by AIC.

```
Call: model.avg(object = dredged[dredged$delta < 4, ])

Component model call:
::(formula = log(p95) ~ <6 unique rhs>, data = moddat, REML = F, na.action = na.fail)
     lme4(formula = log(p95) ~ <6 unique rhs>, data = moddat, REML = F, na.action = na.fail)
     lmer(formula = log(p95) ~ <6 unique rhs>, data = moddat, REML = F, na.action = na.fail)

Component models:
    df logLik  AICc delta weight
14   6 -27.09 68.22  0.00   0.41
1    5 -29.05 69.52  1.30   0.21
134  7 -26.85 70.49  2.27   0.13
124  7 -27.02 70.84  2.62   0.11
13   6 -28.85 71.75  3.53   0.07
12   6 -29.00 72.05  3.83   0.06
```

```
Term codes:
       LineWidth   scale(Time_hr) scale(Time_hr^2)         SeedType
               1                2                3                4

Model-averaged coefficients:
(full average)
                  Estimate Std. Error Adjusted SE z value Pr(>|z|)
(Intercept)       0.318977   0.125480    0.128563   2.481   0.0131 *
LineWidthWide     1.476097   0.196857    0.202690   7.283   <2e-16 ***
LineWidthNarrow   1.271049   0.124749    0.128431   9.897   <2e-16 ***
SeedTypeTypha    -0.144525   0.136707    0.138397   1.044   0.2964
scale(Time_hr^2)  0.012064   0.046122    0.047140   0.256   0.7980
scale(Time_hr)    0.005455   0.039586    0.040670   0.134   0.8933

(conditional average)
                 Estimate Std. Error Adjusted SE z value Pr(>|z|)
(Intercept)       0.31898    0.12548     0.12856   2.481   0.0131 *
LineWidthWide     1.47610    0.19686     0.20269   7.283   <2e-16 ***
LineWidthNarrow   1.27105    0.12475     0.12843   9.897   <2e-16 ***
SeedTypeTypha    -0.22089    0.10814     0.11138   1.983   0.0473 *
scale(Time_hr^2)  0.05960    0.08761     0.09025   0.660   0.5090
scale(Time_hr)    0.03178    0.09107     0.09381   0.339   0.7348
---
Signif. codes:  0 '***' 0.001 '**' 0.01 '*' 0.05 '.' 0.1 ' ' 1

Relative variable importance:
                     LineWidth SeedType scale(Time_hr^2) scale(Time_hr)
Importance:          1.00      0.65     0.20             0.17
N containing models:    6         3        2                2
```

Construction of the final models

Step 4 – Choose a final model structure

The stepwise AIC and the dredging result in very similar model selections. As well, the selection process for p50 and p95 also produce similar results. In the stepwise AIC for p50 and p95, both time variables are dropped from the model, as is SeedType from the p50 model, while they are all retained in the top dredged models. Given that SeedType is retained in the p95 models and given that both variables (SeedType and Time) test specific *a-priori* hypotheses, we opted to keep them in the final model.

The final model took the structure:

$$\textbf{\textit{Dispersal Distance} \sim \textit{Line Width} + \textit{Seed Type} + \textit{Time} + \textit{Time}^2 + (1|\textit{Site})}$$

Step 5 – Fit the final models

For each response, p50 and p95 distances, we built the final model based on the above model selection and calculate the variance explained by the fixed effects (r^2_m) and the full model (r^2_c). We also test the assumptions of the mixed modelling approach, including testing for normality of random effects with a Shapiro-Wilk test (*shapiro.test* command from the base *stats* package for R), visually assessing the homogeneity of residuals by plotting model residuals against fitted values using the *plot.lme* command from the *nlme* package for R[10] and normality of residuals with quantile-quantile plots using the *qqnorm* command from the base *stats* package for R. We assessed the influence of individual data points with plots of Cook's distance and DFBETAS (standardised difference of the betas when individual observations are removed, in units of SE of the parameter estimate) using the *influence* command from the *influence.ME* package for R[11]. Acceptable Cook's distances have been suggested as < 1 or < 4/n, where n is the number of units (< 0.33 in our case) and acceptable levels of DFBETAS have been suggested as < 1 or < 2/√n, where n is the number of units (< 0.58 in our case). However, it can be just as effective to examine the values graphically for visual outliers that may be exerting greater influence over the model than other points.

50th percentile distances (p50):

Final model

```
Formula: log(p50) ~ LineWidth + SeedType + scale(Time_hr) + scale(Time_hr^2) + (1 | Site_No)
   Data: moddat

     AIC      BIC   logLik deviance df.resid
    88.2    103.2    -36.1     72.2       40

Scaled residuals:
    Min      1Q  Median      3Q     Max
-2.2983 -0.5654  0.1123  0.7049  1.5975

Random effects:
 Groups   Name        Variance Std.Dev.
 Site_No  (Intercept) 0.1108   0.3329
 Residual             0.1963   0.4430
Number of obs: 48, groups:  Site_No, 12

Fixed effects:
                 Estimate Std. Error      df t value Pr(>|t|)
(Intercept)       -0.4580     0.1471 27.1500  -3.113  0.00433 **
LineWidthWide      1.2245     0.2337 45.4200   5.238 4.05e-06 ***
LineWidthNarrow    1.1154     0.1485 41.0900   7.512 3.11e-09 ***
SeedTypeTypha     -0.2152     0.1279 35.5700  -1.683  0.10114
scale(Time_hr)    -0.2471     0.3823 19.4100  -0.646  0.52568
scale(Time_hr^2)   0.3239     0.3630 25.4600   0.892  0.38056
---
Signif. codes:  0 '***' 0.001 '**' 0.01 '*' 0.05 '.' 0.1 ' ' 1
```

Variance explained

```
      R2m       R2c
0.5301863 0.6997020
```

Effects plots

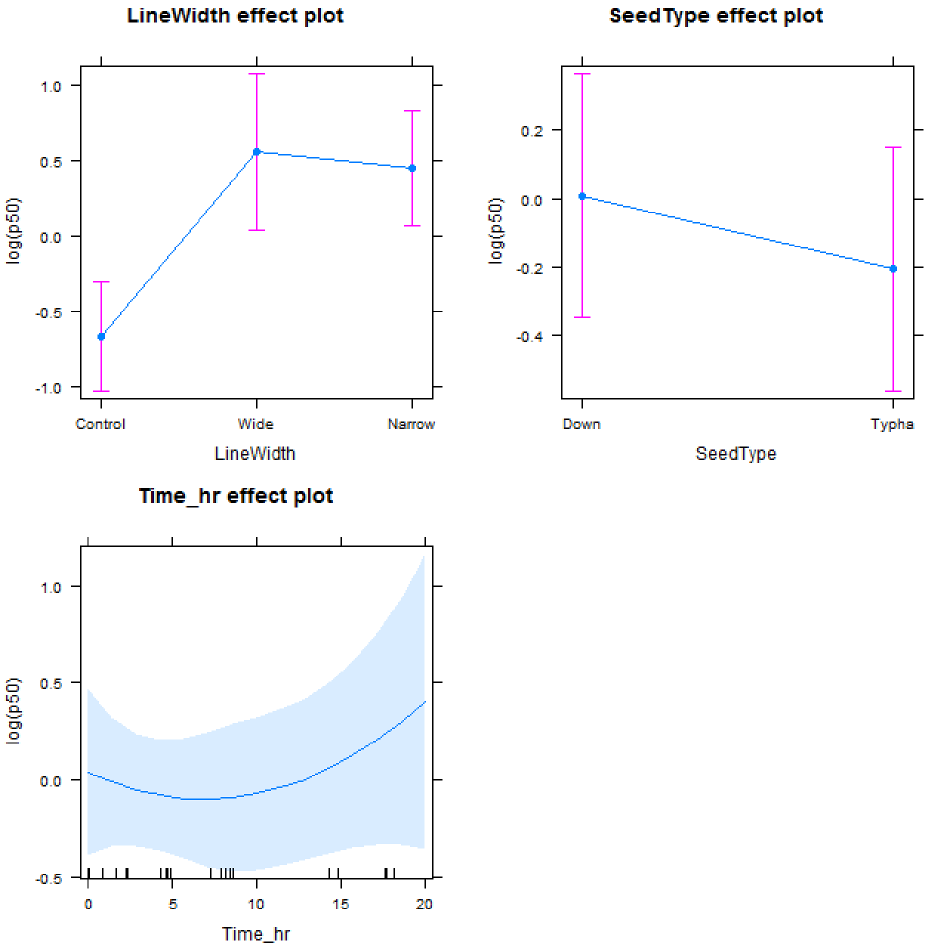

Normality of random effects

```
        Shapiro-Wilk normality test
data:  as.numeric(ranef(d.mixmod)$Site_No[[1]])
W = 0.96139, p-value = 0.8034
```

Homogeneity and normality of residuals

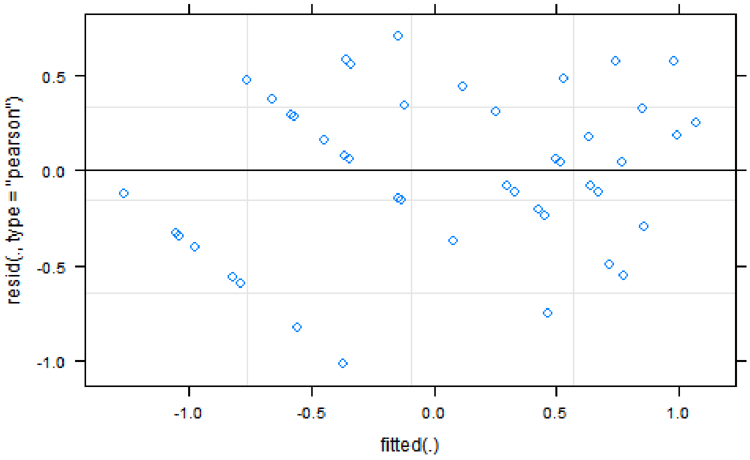

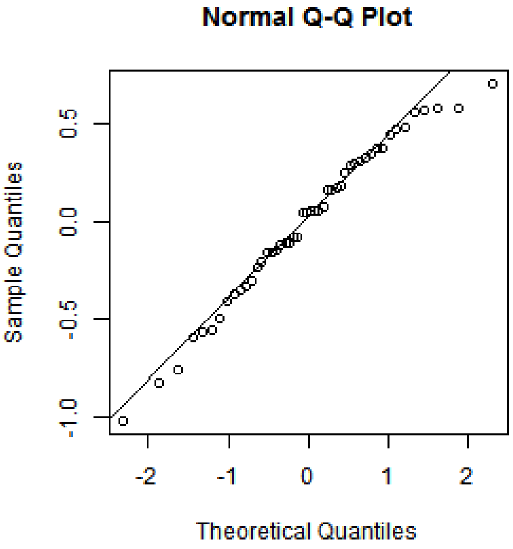

Leverage

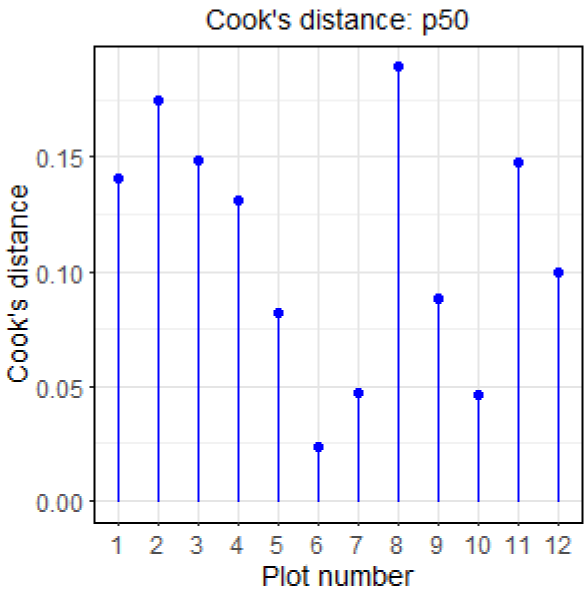

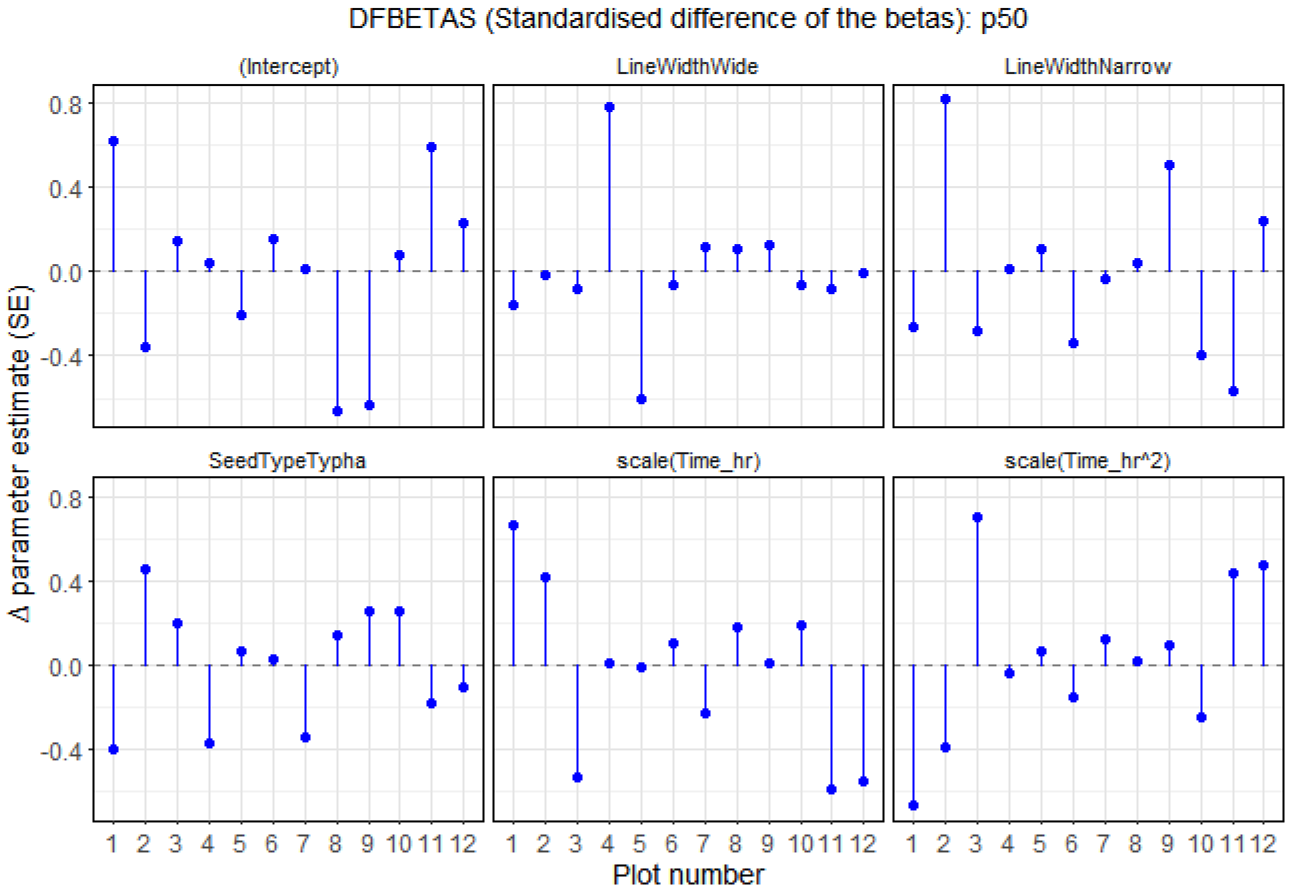
DFBETAS (Standardised difference of the betas): p50
(Intercept)
LineWidthWide
LineWidthNarrow
SeedTypeTypha
scale(Time_hr)
scale(Time_hr^2)
Δ parameter estimate (SE)
0.8
0.4
0.0
-0.4
1 2 3 4 5 6 7 8 9 10 11 12
Plot number

95th percentile distances (p95):

Final model

```
Formula: log(p95) ~ LineWidth + SeedType + scale(Time_hr) + scale(Time_hr^2) + (1 | Site_No)
   Data: moddat

     AIC      BIC   logLik deviance df.resid
    68.7     83.7    -26.4     52.7       40

Scaled residuals:
    Min      1Q  Median      3Q     Max
-1.7292 -0.7654  0.1120  0.6610  2.2636

Random effects:
 Groups   Name        Variance Std.Dev.
 Site_No  (Intercept) 0.05675  0.2382
 Residual             0.13765  0.3710
Number of obs: 48, groups:  Site_No, 12

Fixed effects:
                 Estimate Std. Error      df t value Pr(>|t|)
(Intercept)        0.3527     0.1159 30.1500   3.044  0.00481 **
LineWidthWide      1.4894     0.1925 46.3900   7.736 6.89e-10 ***
LineWidthNarrow    1.2786     0.1233 41.1300  10.366 4.87e-13 ***
SeedTypeTypha     -0.2209     0.1071 35.3300  -2.062  0.04658 *
scale(Time_hr)    -0.2960     0.2956 18.3000  -1.001  0.32968
scale(Time_hr^2)   0.3292     0.2838 23.1500   1.160  0.25783
---
Signif. codes:  0 '***' 0.001 '**' 0.01 '*' 0.05 '.' 0.1 ' ' 1
```

Variance explained

```
      R2m       R2c
0.7070020 0.7925336
```

Effects plots

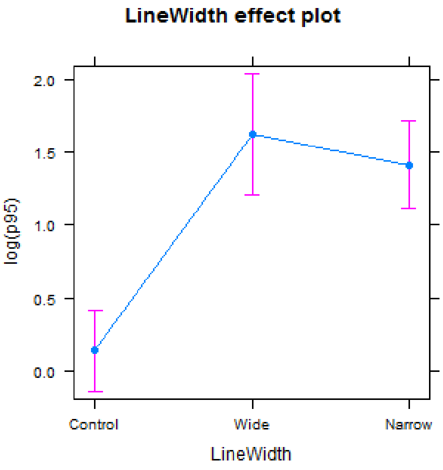

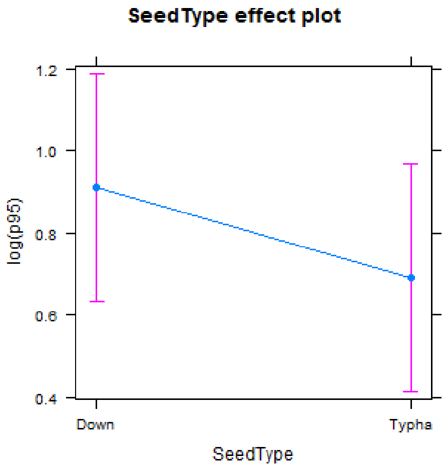

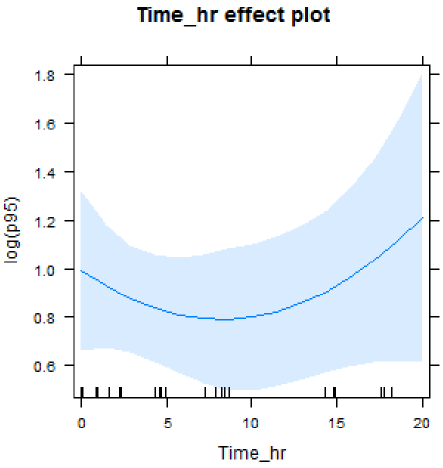

Normality of random effects

```
        Shapiro-Wilk normality test
data:  as.numeric(ranef(d.mixmod)$Site_No[[1]])
W = 0.96845, p-value = 0.8939
```

Homogeneity and normality of residuals

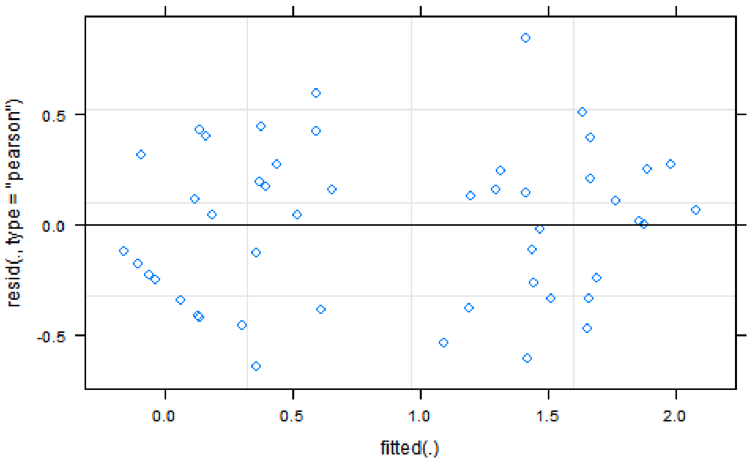

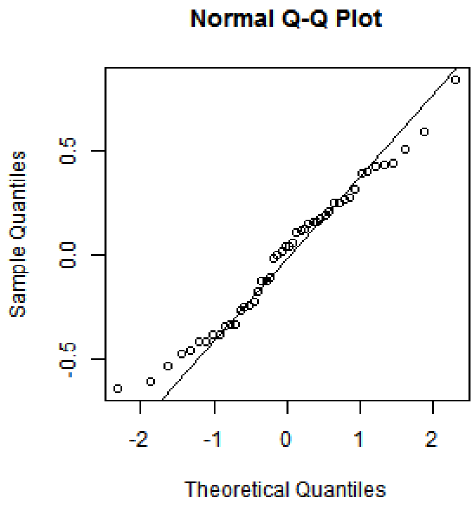

Leverage

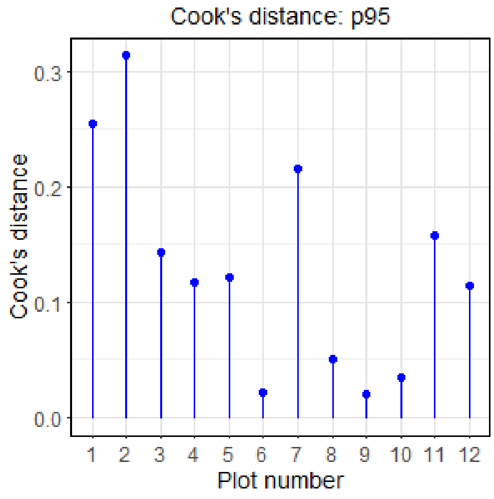

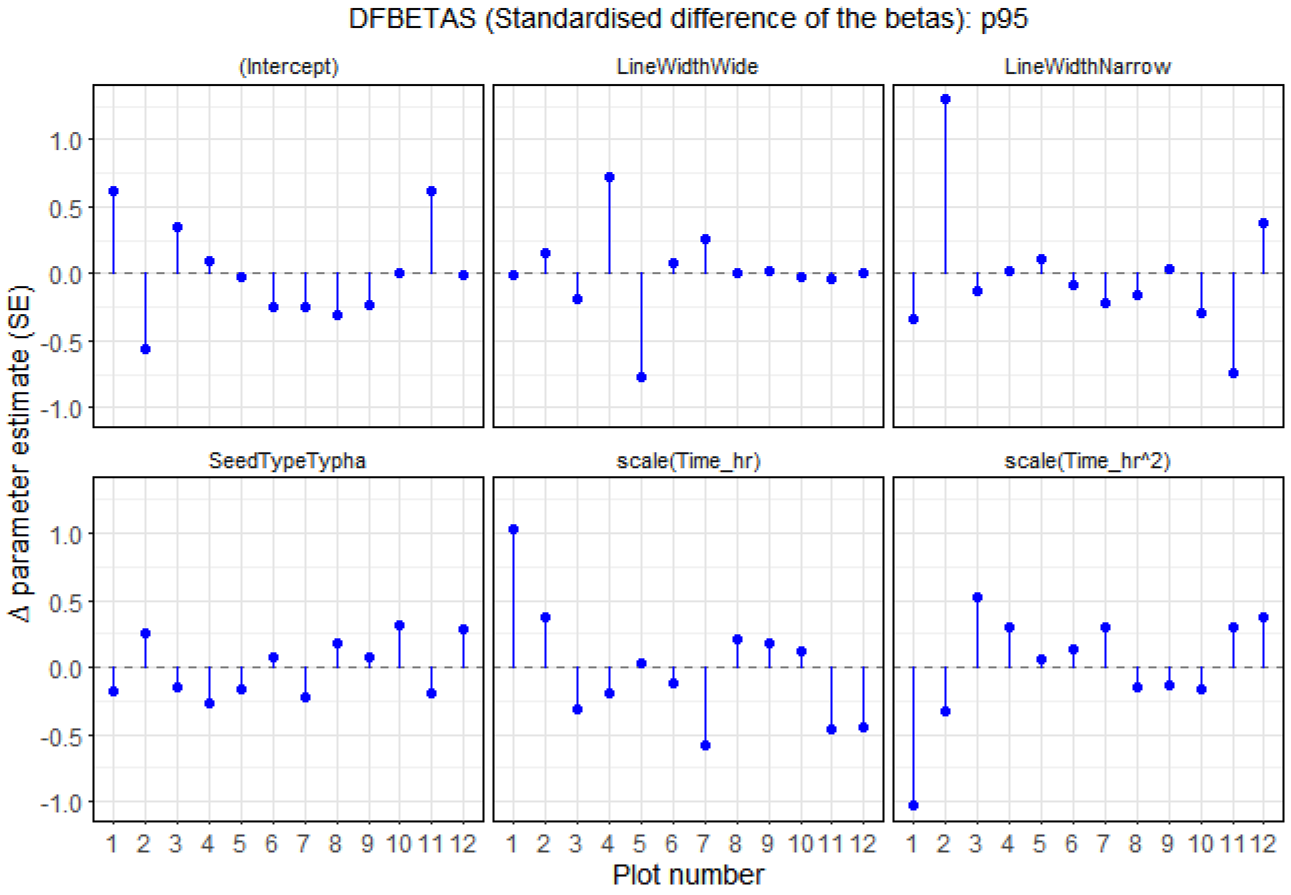
DFBETAS (Standardised difference of the betas): p95
(Intercept)
LineWidthWide
LineWidthNarrow
SeedTypeTypha
scale(Time_hr)
scale(Time_hr^2)
Δ parameter estimate (SE)
Plot number

Appendix 2: LMMs with wind speed

Due to high collinearity between wind speed and the categorical line width variable, measured wind speed was removed from our LMMs (**Table S2, Figure S1, Appendix 1**). However, to demonstrate the clear effect of wind speed on dispersal and to demonstrate the model fit with wind variables, we re-ran the LMMs using the measured wind speed instead of the categorical line width. For both p50 and p95 distances, linear and quadratic wind speed variables were significant in the models. In both models, the AIC was higher and variance explained was lower in wind speed models than in their counterpart line width models (**Appendix 1**).

50th percentile distance

```
Linear mixed model fit by maximum likelihood t-tests use Satterthwaite approximations to
  degrees of freedom [lmerMod]
Formula: log(p50) ~ scale(Wind_kph) + scale(Wind_kph^2) + SeedType + scale(Time_hr) +
    scale(Time_hr^2) + (1 | Site_No)
   Data: moddat

     AIC      BIC   logLik deviance df.resid 
    99.2    114.2    -41.6     83.2       40 

Scaled residuals: 
     Min       1Q   Median       3Q      Max 
-1.83026 -0.60980  0.00744  0.68955  2.32749 

Random effects:
 Groups   Name        Variance Std.Dev.
 Site_No  (Intercept) 0.05894  0.2428  
 Residual             0.28507  0.5339  
Number of obs: 48, groups:  Site_No, 12

Fixed effects:
                  Estimate Std. Error      df t value Pr(>|t|)    
(Intercept)         0.1133     0.1296 22.7400   0.874  0.39110    
scale(Wind_kph)     1.2885     0.2765 47.8900   4.659 2.55e-05 ***
scale(Wind_kph^2)  -0.8662     0.2878 46.3400  -3.010  0.00422 ** 
SeedTypeTypha      -0.2152     0.1541 33.2900  -1.396  0.17182    
scale(Time_hr)     -0.1808     0.3666 14.1100  -0.493  0.62949    
scale(Time_hr^2)    0.2531     0.3587 16.9500   0.705  0.49013    
---
Signif. codes:  0 ‘***’ 0.001 ‘**’ 0.01 ‘*’ 0.05 ‘.’ 0.1 ‘ ’ 1

      R2m       R2c 
0.4596798 0.5522481 
```

95th percentile distance

```
Formula: log(p95) ~ scale(Wind_kph) + scale(Wind_kph^2) + SeedType + scale(Time_hr) +
    scale(Time_hr^2) + (1 | Site_No)
   Data: moddat

     AIC      BIC   logLik deviance df.resid
    85.2    100.2    -34.6     69.2       40

Scaled residuals:
    Min      1Q  Median      3Q     Max
-1.6332 -0.7226 -0.1726  0.6865  2.7743

Random effects:
 Groups   Name        Variance Std.Dev.
 Site_No  (Intercept) 0.009934 0.09967
 Residual             0.238068 0.48792
Number of obs: 48, groups:  Site_No, 12

Fixed effects:
                  Estimate Std. Error      df t value Pr(>|t|)
(Intercept)         1.0183     0.1037 33.0000   9.823 2.53e-11 ***
scale(Wind_kph)     1.5975     0.2390 47.9400   6.684 2.26e-08 ***
scale(Wind_kph^2)  -1.1131     0.2428 43.3200  -4.584 3.85e-05 ***
SeedTypeTypha      -0.2209     0.1409 35.7900  -1.568    0.126
scale(Time_hr)     -0.2589     0.2766 15.2700  -0.936    0.364
scale(Time_hr^2)    0.3025     0.2745 17.1900   1.102    0.286
---
Signif. codes:  0 '***' 0.001 '**' 0.01 '*' 0.05 '.' 0.1 ' ' 1

      R2m       R2c
0.6253681 0.6403750
```

Comparison of AIC and conditional variance explained (r^2_m) for wind speed and line width models:

	p50		p95	
	AIC	r^2_m	AIC	r^2_m
Wind speed	99.2	0.46	85.2	0.63
Line width	88.2	0.53	68.7	0.71

References

1. Brandt, J. P. The extent of the North American boreal zone. *Environ. Rev.* **17,** 101–161 (2009).
2. Efroymson, M. A. Multiple Regression Analysis. in *Mathematical Methods for Digital Computers* (eds. Ralston, A. & Wilf, H. S.) 191–203 (John Wiley & Sons, 1960).
3. Barton, K. *MuMIn: Multi-Model Inference, v1.13.4, http://CRAN.R-project.org/package=MuMIn*. (2013).
4. Burnham, K. P. & Anderson, D. R. *Model Selection and Multimodel Inference: A Practical Information-Theoretic Approach*. (Springer-Verlag, 2002).
5. Symonds, M. R. E. & Moussalli, A. A brief guide to model selection, multimodel inference and model averaging in behavioural ecology using Akaike's information criterion. *Behav. Ecol. Sociobiol.* **65,** 13–21 (2011).
6. Bates, D., Mächler, M., Bolker, B. & Walker, S. C. Fitting linear mixed-effects models using lme4. *J. Stat. Softw.* **67,** 1–48 (2015).
7. Fox, J. Effect displays in R for generalised linear models. *J. Stat. Softw.* **8,** 1–27 (2003).
8. Melis, C. *et al.* Predation has a greater impact in less productive environments: variation in roe deer, *Capreolus capreolus*, population density across Europe. *Glob. Ecol. Biogeogr.* **18,** 724–734 (2009).
9. Arnold, T. W. Uninformative parameters and model selection using Akaike's information criterion. *J. Wildl. Manag.* **74,** 1175–1178 (2010).
10. Pinheiro, J., Bates, D., DebRoy, S. & Sarkar, D. *nlme: Linear and Nonlinear Mixed Effects Models. R package version 3.1-131. https://CRAN.R-project.org/package=nlme*. (2017).
11. Nieuwenhuis, R., Te Grotenhuis, M. & Pelzer, B. influence.ME: tools for detecting influential data in mixed effects models. *R J.* **4,** 38–47 (2012).
